# Identification of Aroma Composition and Key Odorants Contributing to Aroma Characteristics of White Teas

**DOI:** 10.3390/molecules25246050

**Published:** 2020-12-21

**Authors:** Qin-Cao Chen, Yin Zhu, Han Yan, Mei Chen, Dong-Chao Xie, Meng-Qi Wang, De-Jiang Ni, Zhi Lin

**Affiliations:** 1 Tea Research Institute, Chinese Academy of Agricultural Sciences, No. 9 Meiling South Road, West lake District, Hangzhou 310008, China; chenqincao@jxau.edu.cn (Q.-C.C.); zhuy_scu@tricaas.com (Y.Z.); yanhan@tricaas.com (H.Y.); chenxiaomei88520@163.com (M.C.); superxie@tricaas.com (D.-C.X.); lfwangmengqife@126.com (M.-Q.W.); 2 College of Agriculture, Jiangxi Agricultural University, No. 1101 Zhimin Avenue, Qingshan Lake District, Nanchang 330045, China; 3 College of Horticulture and Forestry Science, Huazhong Agricultural University, No. 1 Shizishan Street, Hongshan District, Wuhan 430070, China

**Keywords:** aroma characteristic, GC × GC–TOFMS, multivariate statistical analysis, sensory evaluation, white tea

## Abstract

The identification of aroma composition and key odorants contributing to aroma characteristics of white tea is urgently needed, owing to white tea’s charming flavors and significant health benefits. In this study, a total of 238 volatile components were identified in the three subtypes of white teas using headspace solid-phase microextraction (HS-SPME) combined with comprehensive two-dimensional gas chromatography–time-of-flight mass spectrometry (GC × GC–TOFMS). The multivariate statistical analysis demonstrated that the contents of 103 volatile compounds showed extremely significant differences, of which 44 compounds presented higher contents in Baihaoyinzhen and Baimudan, while the other 59 compounds exhibited higher contents in Shoumei. The sensory evaluation experiment carried out by gas chromatography–olfactometry/mass spectrometry (GC–O/MS) revealed 44 aroma-active compounds, of which 25 compounds were identified, including 9 alcohols, 6 aldehydes, 5 ketones, and 5 other compounds. These odorants mostly presented green, fresh, floral, fruity, or sweet odors. Multivariate analyses of chemical characterization and sensory evaluation results showed that high proportions of alcohols and aldehydes form the basis of green and fresh aroma characteristic of white teas, and phenylethyl alcohol, γ-Nonalactone, trans-β-ionone, trans-linalool oxide (furanoid), α-ionone, and cis-3-hexenyl butyrate were considered as the key odorants accounting for the different aroma characteristics of the three subtypes of white tea. The results will contribute to in-depth understand chemical and sensory markers associated with different subtypes of white tea, and provide a solid foundation for tea aroma quality control and improvement.

## 1. Introduction

White tea, one of six kinds of teas in China, originates from, and is mainly produced in, the northeast of Fujian province, and has received increasing attention in China owing to its charming flavors and significant health benefits [1]. Based on the tenderness level of fresh tea leaves, white teas are generally classified as three subtypes: Baihaoyinzhen (BHYZ, also called silver needle, bud only), Baimudan (BMD, also called white peony, bud with one or two leaves), and Shoumei (SM, more than two leaves with or without bud), ranking from high to low quality degree [2]. Numerous studies indicated that the main taste compounds in white teas are catechins and its polymers, free amino acids, caffeine, flavone, and flavonol glycosides, soluble sugars, phenolic acids, nucleosides, and nucleotides [2,3,4,5,6].

Aroma is also an important factor that determines the quality of teas and affects the choice of consumers. The unique and simple processing gives white tea a green, fresh, or pekoe aroma, which gains widespread popularity among different customers. The aroma profiles of white teas have been increasingly studied [7,8], and the enantiomeric distributions of important volatile compounds [9] and effects of rapid aging technology on the aroma quality of white tea [10] were also recently investigated. Unlike green tea and black tea, which have rich volatile Maillard reaction products [11,12], and Pu-erh tea, which has rich methoxy-phenolic compounds derived from microorganism activities [13], white tea is just rich in volatile compounds, inherent to fresh leaves, for instance aldehydes and alcohols [7]; and hexanal, (E)-2-hexenal, linalool oxide II, linalool, (E)-geraniol, phenylethyl alcohol, benzaldehyde and methyl salicylate, etc., were reported as the main volatile compounds [7,8]. Compared to other kinds of teas processed from the same tea cultivar, the total volatile content of white tea was less than black tea, but more than other teas [14]. However, due to the shortcomings of gas chromatograph mass spectrometry (GC-MS), many vital volatile compounds with low contents may be omitted in these past studies. Fortunately, more comprehensive aroma profiles of white tea were expected by using a superior comprehensive two-dimensional gas chromatography–time-of-flight mass spectrometry (GC × GC–TOFMS) technology, which instantly attracted widespread attention in the field of food flavor analysis, owing to its more powerful performance on resolving power, peak capacity, and structured chromatogram [12,15].

On the other hand, more than 600 volatile compounds have been reported in teas, most of which appear in low or even trace concentration levels [16]. As odor thresholds of volatile compounds vary widely, volatile compounds in low levels frequently have more influence on the aroma quality of teas than those in high levels [17]. Moreover, different volatile compounds display various odors, pleasant, unpleasant, pungent, or flat. Thus, it is difficult and insufficient to measure the contributions of individual volatile compound to the formation of tea aroma purely by chemical characterization, and the assistant sensory technology is necessary to be applied to directly provide the detail aroma performance of each separated volatile component of teas. In recent years, gas chromatography–olfactometry/mass spectrometry (GC–O/MS) has been widely used as an effective sensory scientific technique to identify key odorants accounting for aroma characteristics of teas and other foods [17,18,19]. However, unlike various studies focused on key odorants contributing to aroma characteristic of green teas [20,21], black teas [11,22], oolong teas [23], and dark teas [13,24], the above research in white teas have less systematically investigated, which greatly limits the scientific cognition for consumers. Therefore, the target of this study was to find out more comprehensive information about aroma composition of different subtypes of white teas, and to explore key odorants accounting for aroma characteristics of corresponding types of white tea simultaneously through an improved chemical characterization (GC × GC–TOFMS), and the intuitive sensory evaluation approach (GC–O/MS).

## 2. Results

### 2.1. The Aroma Profile in Different Subtypes of White Teas

Among the collected 17 white tea samples, most samples have good aroma quality except for samples no. 10 and 17, which received low scores (<80) and poor evaluations; thus, samples no. 10 and 17 were removed in further analysis (Supplementary Table S1).

A total of 586 compounds were primarily identified as common volatile compounds after peak alignment (the signal to noise ratio was set to 50), and then the comparison of retention index (RI) and verification of standards resulted in 238 reliable volatile compounds, including 17 alkanes, 28 alkenes, 22 aromatic hydrocarbons, 27 alcohols, 45 aldehydes, 39 ketones, 29 esters, 3 acids, 15 oxygen heterocyclic compounds, 10 nitrogen compounds, 1 sulfur compound, and 2 other compounds (Supplementary Table S2). Notably, two 3,5-octadien-2-one isomers were detected in this study. On account of the existence of two carbon-carbon double bonds, 3,5-octadien-2-one has four structural isomers, while the absolute configuration of 3,5-octadien-2-one is unable to be determined due to lack of corresponding standards. Therefore, the two 3,5-octadien-2-one isomers were referred to as isomer 1 and isomer 2. Similar to 3,5-octadien-2-one, two 2,4-heptadienal isomers were also referred to as isomer 1 and isomer 2.

**Figure 1:**
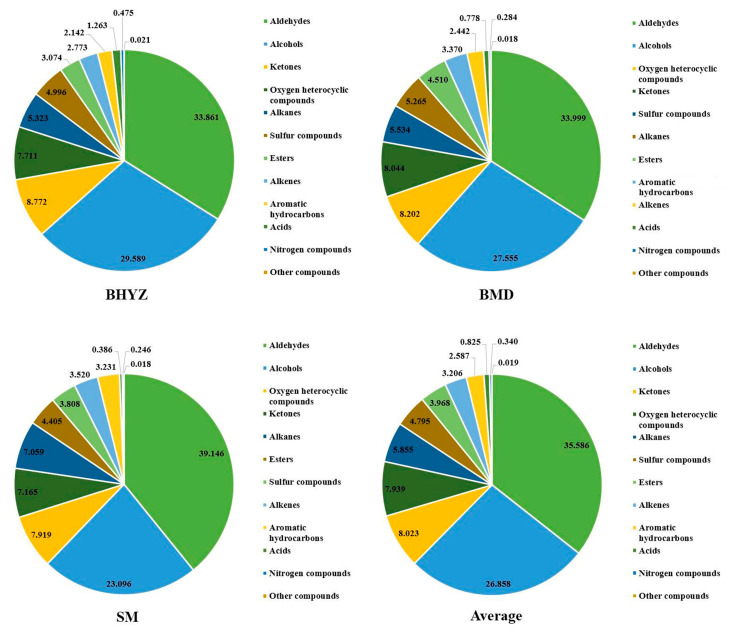
The content level distributions (%) of different kinds of volatile compounds in white teas.

The content distributions of aroma composition in white teas were shown in Figure 1. Overall, the composition of volatile compounds in three subtypes of white teas was similar with slight differences in the exact amounts. It was common that the aldehydes were the richest volatile compounds in white teas (average of 35.586%), following by the alcohols (average of 26.858%), and the total content of aldehydes and alcohols was more than 60%. Although numerous numbers of ketones, esters, and alkenes were observed, the total contents of these were under 9% in each group of white tea. Alkanes, aromatic hydrocarbons, and sulfur compounds ranged from 2% to 7%. Conversely, acids, nitrogen compounds and other compounds showed extremely low contents.

Among the 238 volatile compounds, 30 volatiles with high relative content are listed in Table 1. These 30 volatiles mainly comprise of aldehydes, alcohols, ketones, and several other compounds. Benzaldehyde presented the highest content in white teas with average proportion of 148.4‰, followed by linalool (72.2‰), phenylethyl alcohol (72.0‰), (E)-2-hexenal (65.3‰), dimethyl sulfide (47.4‰), 2-pentyl-furan (47.0‰), geraniol (43.5‰), etc., which mainly displayed natural green, floral, fruity, or sweet odors [16,25]. The results of Tukey s-b(K) test showed that the contents of most of volatile compounds in BHYZ and BMD, such as benzaldehyde, phenylethyl alcohol, dimethyl sulfide, geraniol, hexanal, and benzyl alcohol, etc., were significantly higher than them in SM. High relative contents of these main volatile compounds may confer on BHYZ and BMD a better aroma quality.

### 2.2. Screening of Differential Volatile Compounds among the Three Subtypes of White Teas

**Figure 2:**
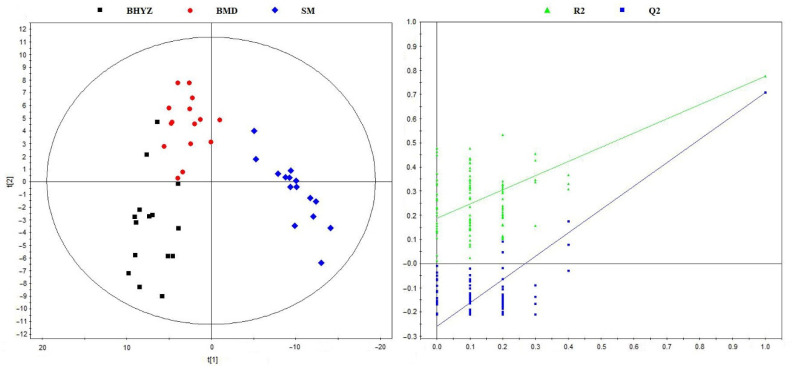
(**a**) The partial least squares discrimination analysis (PLS-DA) score plot (R^2^X = 0.637, R^2^Y = 0.958, Q^2^ = 0.855); (**b**): cross-validation plot of the PLS-DA model with 100 permutation tests (R^2^ = 0.186, Q^2^ = −0.262).

To obtain an overview of the distribution differences of volatile compounds among the three subtypes of white teas, the partial least squares discrimination analysis (PLS-DA) approach was applied based on the peak areas of 238 identified volatile compounds. As shown in Figure 2a, BHYZ, BMD, and SM samples were well discriminated, indicating the presence of obvious difference in the distribution of volatile compounds among BHYZ, BMD and SM. The cross-validation analysis of above PLS-DA model showed that this model was reliable (Figure 2b, R^2^ = 0.187, Q^2^ = −0.259).

**Figure 3:**
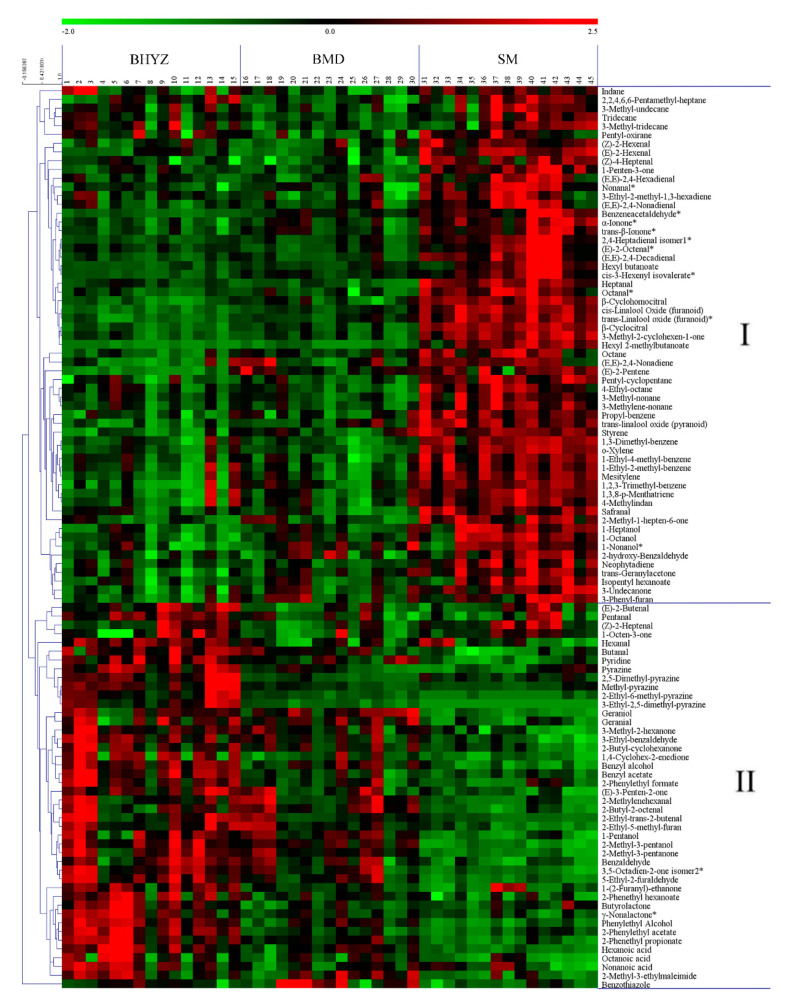
Heat map of the contents of significantly differential volatile compounds in white teas. The data were UV-scaled.

A total of 103 volatile compounds were determined as the important differential volatile compounds based on variable importance in the projection values with a threshold of 1.0 and P value less than 0.01 (Tukey s-b(K) test). As shown in Figure 3, similar content distribution of above 103 compounds was observed in BHYZ and BMD samples, while significant differences existed between BHYZ/BMD and SM samples. These volatile compounds could be clearly divided into two primary classes: 59 volatile compounds in class I, and 44 volatile compounds in class II. The compounds in class I exhibited higher content levels in SM samples than in BHYZ and BMD samples. In contrast, the compounds in class II presented higher content levels in BHYZ and BMD samples than in SM samples.

Our sensory evaluation results (Supplementary Table S1) and long-term sensory evaluation experiences of white teas indicated that more pleasant aroma flavor, such as green, sweet, floral, or pekoe scent, were emitted by BHYZ and BMD, while harsh, woody, or herbal odor were more prominent from SM. Therefore, the above compounds were considered as closely correlating with the aroma quality of white teas. The fatty-acid derived volatiles and some other compounds, such as (E)-2-hexenal, 1-penten-3-one, nonanal, (E,E)-2,4-hexadienal, (E)-2-octenal, heptanal, 1-heptanol, 1-octanol, α-ionone, and trans-β-ionone, which generally presented green, fresh, fatty or woody odor [16,25], could be considered as potential characteristic aroma compositions in SM. Meanwhile, the volatile terpenoids, aromatic alcohols and aromatic esters, including geraniol, geranial, benzyl alcohol, phenylethyl alcohol, 2-phenylethyl formate, benzyl acetate, 2-phenylethyl acetate, 2-phenethyl propionate, and 2-phenethyl hexanoate, which generally showed floral, clean, or pleasant fragrances [16,25], could be considered as potential characteristic aroma components in BHYZ and BMD. However, owing to diverse odor thresholds presented by volatile compounds, some key aroma-active compounds might be ignored or misjudged in above analysis. Therefore, the GC–O/MS method was further performed to directly determine the odor characteristics and intensities of aroma-active compounds in the three subtypes of white teas.

### 2.3. Identification of Aroma-Active Compounds in White Teas Using GC–O/MS

According to the results of sensory evaluation on white teas (Supplementary Table S1), three representative white tea samples (no. 3, 8, 15) were used for GC–O/MS analysis. A total of 44 aroma-active compounds were recognized in white teas (Table 2, and detailed performance of each assessor is showed in Supplementary Tables S3–S5). These odorants could be classified into four types based on their odor characteristics: odorants in class A mainly presented herbal-like scents, such as green, fresh, or grassy odors; odorants in class B mainly presented pleasant scents, such as floral, fruity, or sweet odors; odorants in class C mainly presented baked scents, such as roasted, caramel-like, or coffee-like odors; and odorants in class D mainly presented unpleasant scents, such as plastic, irritating, or stinky odors.

Among the aroma-active compounds, 25 compounds (9 alcohols, 6 aldehydes, 5 ketones, 2 esters, 2 alkenes, and 1 aromatic hydrocarbon) were ultimately determined. Most of the 25 aroma-active compounds belong to class A and B, and mainly displayed pleasant fragrances, such as green, fresh, floral, fruity or sweet scents. In BHYZ, (E)-3-nonen-1-ol, (E)-2-nonenal, benzene acetaldehyde, and nonanal showed the strongest aroma intensities (3.0), followed by linalool and phenylethyl alcohol (2.6–2.8); notably, β-cymene was only detected in BHYZ although the weakest intensity (1.3) was presented.

The stronger aroma intensities and larger number of aroma-active compounds were observed in BMD than in BHYZ and SM. Nonanal (3.6) and linalool (3.4) presented pleasant and remarkable intensities, followed by benzyl alcohol, benzene acetaldehyde, 3,5-octadien-2-one isomer 2, and 5-ethyl-6-methyl-3-hepten-2-one, etc. (3.0–3.2); analogously, neral (1.7) presented the lowest intensity yet was solely found in BMD.

The lowest number of odorants were identified in SM, and the strongest aroma intensities were contributed by (E)-3-nonen-1-ol (3.6) and nonanal (3.5), followed by other delightful odorants including linalool, geraniol, 1-octen-3-ol, and trans-β-ionone (3.0–3.2); cis-3-hexenyl butyrate (2.3), with an herbal and fresh scent, was only detected in SM.

Unfortunately, the chemical structures of other 19 aroma-active compounds were unable to be determined and were named as unknown 1–19 although significant aroma intensities of them were sniffed (Table 2); this is likely attributed to the extremely low both concentrations and odor thresholds of corresponding volatiles and the imperfection of the current HS/SPME-GC-MS approach. Unlike the similar and pleasant odor characteristics displayed by the identifiable odorants, the unknown compounds, mainly belonging to classes D, C and B, presented several novel odors, such as roasted, caramel-like, and stinky odors. Moreover, unknown 10 (2.8), 9 (3.3), and 6 (4.0) presented the strongest intensities in BHYZ, BMD, and SM, respectively. It is worth noting that a powder-like odor, similar to the pekoe scents of fresh and tender white teas, was exhibited by unknown 12 and 18 with moderate intensities in BHYZ and BMD, indicating that these two compounds might contribute to the pekoe odor of BHYZ and BMD.

## 3. Discussion

### 3.1. The Comparison of Obtained and Reported Aroma Profiles of White Teas

As mentioned above, alcohols and aldehydes made up large proportions of aroma profile of white teas in this work, which was consistent with our previous study [26]. It has been proven that the contents of aldehydes and alcohols were abundant in fresh tea leaves [26]; besides, as the prolonged withering period is similar to fermentation process to a certain degree [3], in which fatty acids and amino acids are largely degraded to alcohols and aldehydes [16], the alcohols and aldehydes was largely generated during the prolonged withering period [26,27]. It may explain the aldehydes and alcohols were the most abundant volatile compounds in white teas.

Differently, it was reported by Wang’s group that the average content of aldehydes in white teas (BHYZ and BMD) was as low as 2.34% [7]. In addition, the oxygen heterocyclic compounds, especially furan derivatives, showed a relatively high content level of 7.939% in this study compared to previous studies [7,8]. The above inconsistence might be caused by the different sources of white tea samples and improve analytical techniques used in this study.

### 3.2. The Comparison of Obtained and Reported Aroma-Active Compounds in Teas

It was roughly identical to previous reports that fatty acid-derived alcohols, aldehydes, and esters were the main components contribute to herbal-like scents (class A) of teas [16,19]. Among them, (E)-2-nonenal was reported as an important odorant contributing to the aroma characteristic of green tea [20,21] and matcha tea [28]; and hexanal was proven to be an important contributor to the aroma characteristic of green tea [21] and oolong tea [23]. It is worth noting that these odorants presented dual odor characteristics. In addition to green and fresh odor, unpleasant grassy or stinky odor were also displayed by these odorants. It is common that a volatile compound associate with a pleasant odor at low concentration, while present an unpleasant odor at high concentration [29,30]. Therefore, a moderate proportion of odorants in class A would confer on white teas a pleasant green or fresh aroma, while excessively high proportion of odorants in class A may render white teas an unpleasant grassy odor.

The identified odorants in class B mainly consist of volatile terpenoids, amino acid-derived volatiles and carotenoid derived volatiles [16,19]. Unlike the odorants in class A possessed dual odor characteristics, the odorants in class B presented floral, fruity, sweet, or other pleasant odors no matter at low or high concentration. High ratio of odorants in class B may confer on white tea floral or fruity scents. Linalool, geraniol, trans-β-ionone, trans-linalool oxide (furanoid), and phenylethyl alcohol have been previously reported as key aroma-active compounds responsible for the aroma of black teas [11,25] and oolong teas [23]. Trans-β-ionone and α-ionone were proved to be important contributors to woody flavor of Pu-erh teas with a very low odor threshold [13,24], while a woody flavor was not displayed by trans-β-ionone and α-ionone in the present study, which may owe to their low contents in white teas compared with Pu-erh teas. Naphthalene is the sole identified odorants in class D, and presented the unpleasant odor with moderate and high intensity (2.0–3.3).

### 3.3. The Comparison of Total Aroma Intensities of Aroma-Active Compounds among Different Subtypes of White Teas

**Figure 4:**
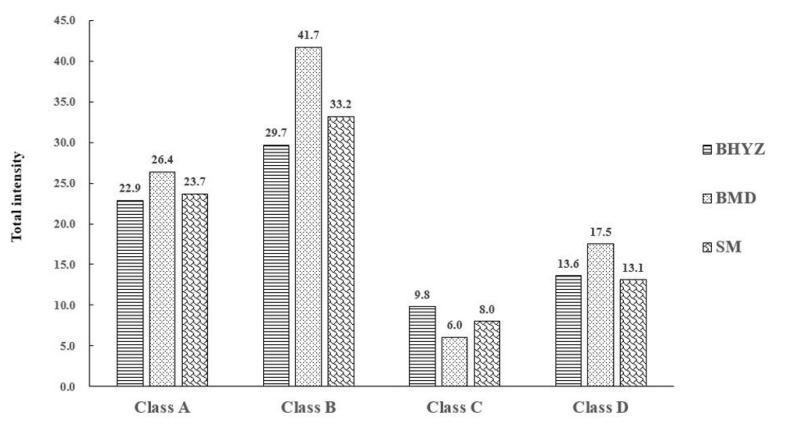
Total intensities of aroma-active compounds belonged to different classes (A: herbal-like odors; B: pleasant (floral, fruity, sweet) odors; C: baked odors; D: unpleasant odors) in three subtypes of white teas.

Furthermore, the aroma intensities of aroma-active compounds in different classifications among different subtypes of white teas were summarized and compared (Figure 4). The order of total aroma intensities among three subtypes of white teas was consistent, which was showed as follows: class B > class A > class D > class C; and the total intensity of class B and class A is notably more than it of class D and class C; thus, high proportion of odorants in class B and A might makes up the foundation of green and fresh aroma of white teas. As noticeable stronger aroma intensities of aroma-active compounds in most classes, especially class B, were observed in BMD compared with BHYZ and SM, in combination with sensory evaluation results of BMD, our findings indicated that some odorants in class B might be important contributors of pekoe flavor. Meanwhile, the synergistic and inhibitory effects between each odorant were also considerable, and thus related studies are currently ongoing to better understand the characteristics of white tea aroma.

### 3.4. The Comparison of the Results Obtained from GC × GC–TOFMS and GC–O/MS

**Figure 5:**
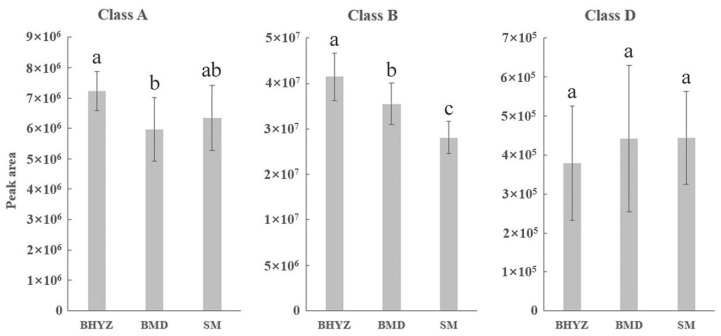
Comparison of total peak area of the 25 identified odorants in different odor classes among BHYZ, BMD, and SM. Note: different letters (a, b, ab, c) above the histograms represent the statistic difference (*p* < 0.05) among different groups.

It is well known that the aroma quality of teas is deeply influenced by the tenderness of fresh tea leaves under the same processing conditions [6], and thus BHYZ generally presents the best aroma quality, following by BMD and SM. Moreover, it is predictable that the aroma quality of a tea is closely correlated with the concentrations of volatiles, especially aroma-active compounds. Hence, it is interesting and necessary to investigate the content distribution of the 25 identified aroma-active compounds with different odor classification among BHYZ, BMD, and SM. As shown in Figure 5, the total contents of class A odorants in BHYZ were the most abundant (BHYZ > SM > BMD), and the total contents of class B odorants were consistent with the tenderness of white teas (BHYZ > BMD > SM), while unpleasant class D odorants presented a reverse trend (SM > BMD > BHYZ), which was in line with expectations. Overall, the higher content levels of odorants in classes A and B and lower content levels of odorants in class D might make a significant contribution to the formation of excellent aroma quality of white teas.

Notably, the alcohols and aldehydes, which mainly presented natural herbal fragrance, were the richest compounds in both GC × GC–TOFMS and GC–O/MS results. Similar with that the methoxy-phenolic compounds and alcohols was suggested to play a vital role in the special flavor of Pu-erh teas [13], it could be proposed that high proportion of aldehydes and alcohols with natural pleasant scents forms the basis of green and fresh aroma characteristic of white teas.

### 3.5. Determination of Key Odorants Accounting for Aroma Characteristics of the Three Subtypes of White Teas

As mentioned above, although various key differential volatile compounds among the three subtypes of white teas have been identified via chemical characterization (GC × GC–TOFMS analysis) combined with multivariate statistical analysis, many of which probably contribute less to the formation of unique aroma quality of the three subtypes of white teas due to their high odor thresholds. As for GC–O/MS analysis, the different sensitivity and subjectivity of panelists resulted in some inevitable deviations and minor differences of aroma intensities between each group of white teas during sensory evaluation. Therefore, it was necessary to comprehensive analysis and verify the results obtained by both chemical characterization and sensory evaluation, and the common identified compounds were considered as the potential key odorants accounting for the aroma characteristics of white teas.

**Figure 6:**
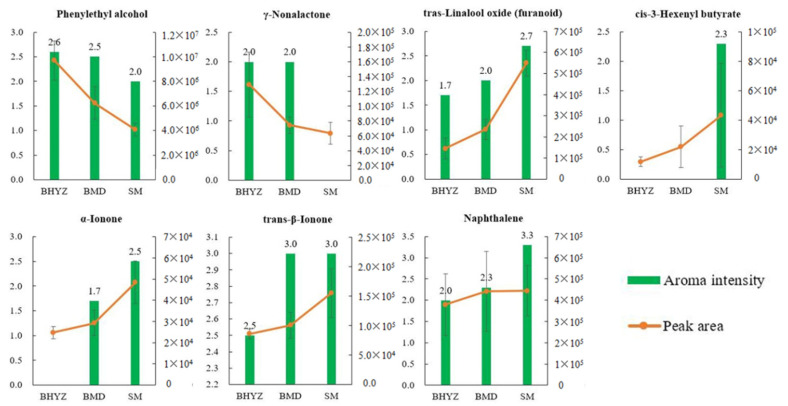
Distribution on the aroma intensity and content of key odorants accounting for the aroma characteristics of white teas.

A total of 14 aroma-active compounds with VIP > 1 in the PLS-DA model were screened, including hexanal, benzyl alcohol, benzeneacetaldehyde, (E)-2-octenal, trans-linalool oxide (furanoid), 3,5-octadien-2-one isomer 2, nonanal, 1-nonanol, phenylethyl alcohol, cis-3-hexenyl isovalerate, geraniol, γ-Nonalactone, α-ionone, and trans-β-ionone. However, probably mainly due to the experimental errors brought by quantitation during the two methods and some differences on the experiment conditions between them, the irrelevant or converse correlations between the distribution of aroma intensities and contents of some potential key odorants among the three subtypes of white teas were observed. Notably, as shown in Figure 6, phenylethyl alcohol, γ-Nonalactone, trans-β-ionone, trans-linalool oxide (furanoid), and α-ionone presented the consistent variation trends in both aroma intensities and relative content levels. Besides, the aroma-active compounds, which only sniffed in one subtype of white tea were also examined, and the exclusive odorants of SM named cis-3-hexenyl butyrate exhibited significant higher content levels in SM samples. Thus, the above six compounds were further determined as the key odorants in the different subtypes of white teas.

Phenylethyl alcohol with floral and sweet scents might play an important role in the formation of excellent aroma quality of BHYZ for its highest content level and aroma intensity among the samples. The pleasant γ-Nonalactone (sweet and cream-like odor), which was unable to be sniffed in SM, but showed equal aroma intensity in BHYZ and BMD, may confer BHYZ and BMD a better aroma quality compared to SM, and it might contribute more in BHYZ owing to the corresponding highest content levels. Trans-β-ionone exhibited the equal and strong aroma intensity in BMD and SM, indicating its great importance in these two subtypes of teas, and it might promote the formation of the woody and harsh odor of white teas for its similar scents presented at a higher concentration [13,24]. In additions, trans-linalool oxide (furanoid), α-ionone, and cis-3-hexenyl butyrate were considered as the key odorants responsible for the aroma characteristics of SM owing to their obviously higher contents and aroma intensities in SM than those in BHYZ and BMD.

## 4. Materials and Methods

### 4.1. Tea Samples

A total of 17 white tea samples, including 5 BHYZ, 6 BMD, and 6 SM, were collected in this study (Supplementary Table S1). All samples were produced in Fuding, Fujian province of China at 2018. For quality assurance, these samples were authenticated and sensory evaluated by the experienced experts from the Tea Research Institute of Chinese Academy of Agricultural Sciences based on the national standard named “White Tea (GB/T 22291-2017)”. Before conducting aroma analysis, tea samples were ground into homogeneous powders using a tube mill (IKA, Staufen, Germany) and then stored at −20 °C.

### 4.2. Chemicals and Reagents

(E)-2-Octenal, octanal, linalool, trans-linalool oxide (furanoid), nonanal, (E,E)-2,4-heptadienal, (Z)-3-nonen-1-ol, (E)-2-nonenal, 1-nonanol, cis-3-hexenyl isovalerate, α-ionone, and trans-β-ionone were purchased from TCI corporation (Tokyo, Japan). The 1-hexanol, 1-octen-3-ol, benzene acetaldehyde, and naphthalene were purchased from Aladdin Corporation (Shanghai, China). The 3,5-octadien-2-one was purchased from AA Blocks Corporation (San Diego, CA, USA). γ-Nonalactone and cis-3-hexenyl butyrate were purchased from Adamas Corporation (Shanghai, China). α-Cedrene was purchased from Heowns Corporation (Tianjin, China). N-alkanes (C8–C40) were purchased from J&K Scientific Corporation (Beijing, China).

### 4.3. Headspace Solid-Phase Microextraction (HS-SPME)

The extraction of volatile compounds has been optimized in our previous work [11,26]. Volatile compounds were extracted by the headspace solid-phase microextraction (HS-SPME) method with a carboxen/polydimethylsiloxane (CAR/PDMS) coating fiber (85 µm; 1 cm; Supelco, Inc., Bellefonte, PA, USA). The parameters were as follows: 1.0 g of tea powder was firstly placed into a 20 mL glass vial, and then 6 mL of boiling deionized water was added; subsequently, the glass vial was immediately sealed with a cover and placed into a thermostatic oscillator, incubating at 60 °C; after 3 min of stabilization, volatile compounds were adsorbed for 60 min using a CAR/PDMS fiber. Finally, volatile compounds were desorbed at 250 °C for 5 min for GC × GC–TOFMS and GC–O analysis.

### 4.4. GC × GC–TOFMS Analysis

The GC-MS system is a LECO Pegasus 4D GC × GC–TOFMS coupled with quad-jet dual stage thermal modulator (LECO Corporation, St. Joseph, MI, USA). The 1st D and 2nd D column are Rxi-5MS MS column (30 m × 250 µm × 0.25 µm; Restek, Bellefonte, PA, USA) and Rxi-17Sil MS column (1.9 m × 100 µm × 0.10 µm; Restek, Bellefonte, PA, USA), respectively.

GC conditions were as follows: the raising temperature procedures for 1st D column were: 50 °C for 2 min and slowly increased to 265 °C at 8 °C /min, then held for 5 min; the raising temperature procedures for 2nd D column were: 55 °C for 2 min, slowly increased to 270 °C at 8 °C /min, then held for 5 min; the helium (99.999%) was used as carrier gas with a constant flow of 1.0 mL/min; the transfer line temperature was 250 °C; the split mode was used with a ratio of 10:1; the modulation period was 5.0 s.

Mass spectrometry was performed at an ionization voltage of 1590 V with a scan range of 33–600 *m*/*z*. The electron impact ionization was –70 eV. The ion source temperature was 220 °C, and the interface temperature was 270 °C.

The GC × GC–TOFMS data were processed using a LECO Chroma TOF software. The 1st D and 2nd D peak width were 25 and 0.1 s, respectively. The minimum S/N was 50, and the minimum similarity match was 750 (maximum of 1000). The integrated peak table of all samples was accomplished using the statistical comparison function in the Chroma TOF software, and the deviations of 1st D retention time (RT) and 2nd D RT were 0.2 min and 0.2 s, respectively. The 1st D Kovats (RI) of volatile compounds were calculated using series of n-alkanes (C8–C40), and the volatile compounds were removed if the RI difference between the experimental 1st D value and reported values was more than 20 [12].

### 4.5. GC–O/MS Analysis

The GC–O/MS analysis is carried out using a GC system (7890B, Agilent Technologies, Santa Clara, CA, USA) coupled with a MS detector (5977B, Agilent Technologies, Santa Clara, CA, USA) and an olfactometry detector (ODP-3, Gerstel GmbH & Co., KG, Germany).

GC conditions: the GC column was a Rxi-5MS column (30 m × 250 µm × 0.25 µm; Restek, Bellefonte, PA, USA); the carrier gas was helium (99.999%) with a constant flow of 1.6 mL/min; the raising temperature procedures for GC column were: 50 ºC for 3 min and slowly increased to 265 °C at 4 °C /min, then held for 5 min; splitless injection mode was applied.

MS conditions: the ionization mode was EI; the ionization voltage was 1353 V; the ion source temperature was 230 °C; the quadrupole temperature was 150 °C; a full scan mode was used with a mass scan of 50–550 *m*/*z*; the electron impact ionization was –70 eV.

Olfactory conditions: the split ratio was 1:1 between MS detector and olfactory detection port; the temperature of transfer line and sniffing port were 260 °C and 230 °C, respectively; the nitrogen (99.99%) was applied as an auxiliary gas with a constant flow of 50 mL/min. The moisture was supplied for GC-sniffing port to prevent the drying of the nose mucous membranes of assessors.

GC–O analysis was performed by five panelists (two males and three females), and each panelist sniffed at least once. Prior to conducting formal GC–O analysis, the panelists were well-trained for more than 90 h to well distinguish different odors by using a series of standards with typical flavors, which have been described in our previous work [11], and they were also trained by two quality control tea samples. The aroma intensities were defined from 1 to 4:1 was weak, 2 was moderate, 3 was strong, and 4 was extremely strong. If a similar odorant was recorded at consistent RTs for at least three panelists, or two panelists with high intensity (equal to or greater than 3), then the record would be accepted and identified via MS, RI and authentic standards. The typical overlay plot of chromatograms of GC-MS and GC–O is shown in Supplementary Figure S1.

### 4.6. Data Processing

The relative contents of volatile compounds were calculated by the formula of A/A *total*, where A represents the peak area of individual volatile compound, and A *total* represents the total peak area of all volatile compounds in an individual sample. Partial least squares discrimination analysis (PLS-DA) was performed using a Simca-P 11.5 software (Umetrics AB Corporation, Umea, Sweden) after UV scaling, and the significance of difference in volatile compounds among groups was calculated by Tukey s-b(K) test using a PASW Statistics software (Version 18.0, USA). The heat map was generated by MultiExperiment Viewer 4.8.1 (Oracle Corporation, Redwood, CA, USA).

## 5. Conclusions

In the present study, the profile of volatile components and key odorants in the three subtypes of white teas were exhaustively investigated using HS-SPME-GC × GC–TOFMS combined with GC–O/MS technology. A total of 238 volatile components were finally identified, of which the aldehydes (average of 35.586%) and alcohols (average of 26.858%) were the richest volatile compounds in white teas. The PLS-DA resulted in 103 extremely significant differential volatile compounds among the three subtypes of white teas. GC–O/MS analysis revealed 44 aroma-active compounds, of which 25 odorants were identified and mainly presented green, fresh, floral, fruity, or sweet scents. The combination of GC × GC–TOFMS and GC–O/MS results indicated that high proportion of aldehydes and alcohols forms the basis of green and fresh aroma characteristic of white teas. Moreover, phenylethyl alcohol, γ-Nonalactone, trans-β-ionone, trans-linalool oxide (furanoid), α-ionone, and cis-3-hexenyl butyrate were considered as the key odorants accounting for the different aroma characteristics of the three subtypes of white teas. The focus of our subsequent work will include deep studies for identification of unknown odorants and investigation of the synergistic and inhibitory effects between odorants.

## Figures and Tables

**Table 1:** Top 30 volatiles with high relative content (‰) in white teas detected by comprehensive two-dimensional gas chromatography–time-of-flight mass spectrometry (GC × GC–TOFMS).

No.	Compounds	BHYZ	BMD	SM	Average
1	Benzaldehyde **	160.4 ± 19.4	156.8 ± 17.7	128.0 ± 7.8	148.4 ± 20.9
2	Linalool	67.3 ± 4.6	71.7 ± 2.8	77.5 ± 9.8	72.2 ± 7.4
3	Phenylethyl alcohol **	101.2 ± 22.9	68.9 ± 16.2	45.9 ± 5.0	72.0 ± 28.0
4	(E)-2-Hexenal **	54.8 ± 9.3	54.8 ± 9.1	86.3 ± 12.6	65.3 ± 18.2
5	Dimethyl sulfide **	49.5 ± 9.7	54.8 ± 12.5	37.8 ± 11.3	47.4 ± 12.7
6	2-Pentyl-furan	42.3 ± 6.7	46.4 ± 12.6	52.3 ± 6.3	47.0 ± 9.3
7	Geraniol **	47.7 ± 10.1	56.1 ± 15.2	26.8 ± 3.6	43.5 ± 16.2
8	Decane	29.2 ± 7.1	30.1 ± 2.4	38.3 ± 6.8	32.5 ± 6.9
9	Methyl salicylate	23.0 ± 6.9	37.3 ± 14.8	34.3 ± 13.8	31.5 ± 13.1
10	Hexanal **	31.5 ± 3.6	25.4 ± 4.7	27.6 ± 7.6	28.1 ± 5.8
11	Benzyl alcohol **	32.4 ± 1.1	26.7 ± 4.8	18.1 ± 3.0	25.7 ± 6.8
12	2-Ethyl-furan **	25.1 ± 5.0	24.1 ± 10.6	16.9 ± 3.3	22.0 ± 7.5
13	Benzene acetaldehyde **	8.9 ± 2.1	17.5 ± 4.5	33.3 ± 14.2	19.9 ± 13.2
14	2-Methyl-butanal	14.1 ± 3.3	14.0 ± 3.7	20.4 ± 9.3	16.1 ± 6.4
15	(Z)-3-Hexen-1-ol	13.1 ± 3.0	13.5 ± 2.0	16.8 ± 5.2	14.5 ± 3.8
16	3,5-Octadien-2-one isomer 1 *	15.3 ± 5.0	15.4 ± 8.2	10.7 ± 3.1	13.8 ± 5.8
17	Nonanal **	16.8 ± 4.2	13.3 ± 4.7	6.8 ± 1.6	12.3 ± 5.5
18	3,5-Octadien-2-one isomer 2 **	9.2 ± 2.2	9.3 ± 4.3	19.4 ± 5.9	12.6 ± 6.4
19	6-Methyl-5-hepten-2-one *	10.4 ± 1.1	8.5 ± 1.7	10.3 ± 1.4	9.7 ± 1.6
20	2-Heptanone **	11.0 ± 2.1	9.4 ± 1.9	7.8 ± 1.5	9.4 ± 2.2
21	Butanal **	12.5 ± 1.8	9.8 ± 1.9	5.9 ± 2.7	9.4 ± 3.5
22	2,4-Heptadienal isomer 2 **	4.7 ± 0.8	6.1 ± 2.8	15.7 ± 9.2	8.8 ± 7.2
23	β-Myrcene **	8.6 ± 1.7	9.4 ± 0.9	7.5 ± 1.1	8.5 ± 1.4
24	Toluene **	6.4 ± 1.5	7.3 ± 1.6	9.5 ± 1.4	7.8 ± 1.9
25	Dodecane	6.9 ± 0.8	7.1 ± 1.5	8.6 ± 1.8	7.5 ± 1.5
26	Hexanoic acid **	11.1 ± 6.0	6.8 ± 1.8	2.8 ± 0.5	6.9 ± 4.9
27	1-Octen-3-ol	7.3 ± 0.7	6.1 ± 1.5	7.1 ± 1.5	6.8 ± 1.3
28	1-Penten-3-ol **	6.6 ± 1.4	6.1 ± 0.9	4.1 ± 0.7	5.6 ± 1.5
29	5-Ethyl-6-methyl-3-hepten-2-one	5.7 ± 1.4	6.0 ± 0.4	5.0 ± 1.2	5.6 ± 1.1
30	Limonene	3.8 ± 0.9	5.4 ± 2.2	5.0 ± 0.6	4.8 ± 1.5

Note: the data are shown as mean value ± SD; * and ** indicates that the compounds presented significant difference (*p* < 0.05) and extremely significant difference (*p* < 0.01) among the three subtypes of white teas, respectively. BHYZ: Baihaoyinzhen, BMD: Baimudan, SM: Shoumei.

**Table 2:** Identified aroma-active compounds in white teas using GC–O/MS.

No.	Compounds	Odor Characteristic	Aroma Intensity ^[1]^			Identification Method ^[2]^	Class ^[3]^
			BHYZ	BMD	SM		
**1**	Hexanal	Green, grassy, fresh	2.0	2.8	2.6	MS, RI	A
**2**	1-Octen-3-ol	Mushroom-like, fresh	2.0	2.5	3.0	MS, RI, STD	A
**3**	β-Cymene	Floral, fruity, rice-like	1.3			MS, RI	B
**4**	Benzyl alcohol	Floral, rose-like, pollen-like	2.0	3.0	2.3	MS, RI	B
**5**	Benzene acetaldehyde	Green, sweet, green lemon-like	3.0	3.2	2.8	MS, RI, STD	B
**6**	(E)-2-Octenal	Stinky, green, sauce-like		2.0	1.7	MS, RI, STD	A
**7**	trans-Linalool oxide (furanoid)	Floral, sweet, roasted	1.7	2.0	2.7	MS, RI, STD	B
**8**	3,5-Octadien-2-one isomer 2	Green, fresh, sweet	2.5	3.2	2.8	MS, RI, STD	A
**9**	Linalool	Floral, fruity, sweet	2.8	3.4	3.2	MS, RI, STD	B
**10**	Nonanal	Floral, fruity, green, fresh	3.0	3.6	3.5	MS, RI, STD	B
**11**	Phenylethyl alcohol	Floral, sweet, rose-like	2.6	2.5	2.0	MS, RI	B
**12**	5-Ethyl-6-methyl-3-hepten-2-one	Stinky, grassy, fresh	2.5	3.0	2.8	MS, RI	A
**13**	(E)-3-Nonen-1-ol	Green, grassy, stinky	3.0	3.2	3.6	MS, RI, STD	A
**14**	(E)-2-Nonenal	Stinky, fresh, cucumber-like	3.0	2.5		MS, RI, STD	A
**15**	1-Nonanol	Green, fresh, grassy	2.0	2.0		MS, RI, STD	A
**16**	Naphthalene	Non-pleasant, irritating	2.0	2.3	3.3	MS, RI, STD	D
**17**	cis-3-Hexenyl butyrate	Fresh, cucumber-like			2.3	MS, RI, STD	A
**18**	Terpineol	Floral, sweet, green, fresh	2.0	2.7	2.0	MS, RI	B
**19**	Neral	Floral, fruity, hay-like		1.7		MS, RI	B
**20**	cis-3-Hexenyl isovalerate	Green, fresh, sweet, floral	2.2	3.0	2.6	MS, RI, STD	A
**21**	Geraniol	Floral, rose-like, fresh	2.2	2.6	3.2	MS, RI	B
**22**	γ-Nonalactone	Sweet, cream-like, floral	2.0	2.0		MS, RI, STD	B
**23**	α-Ionone	Pleasant, cream-like, rose-like		1.7	2.5	MS, RI, STD	B
**24**	trans-β-Ionone	Sweet, cream-like, floral, fruity	3.0	3.0	3.0	MS, RI, STD	B
**25**	cis-Calamenene	Fresh, minty, toothpaste-like	1.7	2.2		MS, RI	A
**26**	Unknown 1	Green, fresh, grassy			2.3		A
**27**	Unknown 2	Floral, coffee-like, sweet	1.8	3.0			B
**28**	Unknown 3	Roasted, milky, sweet	1.8		2.0		C
**29**	Unknown 4	Sweet, roasted, floral, burnt	2.7	3.3	3.0		C
**30**	Unknown 5	Roasted, sweet, cream-like	2.8	2.7	3.0		C
**31**	Unknown 6	Capsule-like, powder-like	1.3				D
**32**	Unknown 7	Non-pleasant, irritating	2.3	2.3	1.8		D
**33**	Unknown 8	Non-pleasant, irritating	2.6	3.0	3.3		D
**34**	Unknown 9	Stinky	2.0		2.7		D
**35**	Unknown 10	Roasted, coffee-like, burnt	2.5				C
**36**	Unknown 11	Non-pleasant, plastic-like		3.0	2.0		D
**37**	Unknown 12	Green, fresh, cucumber-like	2.0				A
**38**	Unknown 13	Non-pleasant, spicy		2.7			D
**39**	Unknown 14	Non-pleasant, powder-like	1.7	2.5			D
**40**	Unknown 15	Floral, fruity, sweet		2.0			B
**41**	Unknown 16	Sweet, fruity, minty, citrus		2.0			B
**42**	Unknown 17	Non-pleasant, irritating	1.7	1.7			D
**43**	Unknown 18	Sweet, caramel-like, floral	2.3	3.3	4.0		B
**44**	Unknown 19	Sweet, honey, caramel-like			2.0		B

Note: ^[1]^ The presented aroma intensity of each compound was the average value obtained from the effective assessors; ^[2]^ STD means the compound was identified by authentic standards; ^[3]^ the classification of odor characteristics of each compound, Class A: herbal-like scents; Class B: pleasant (floral, fruity, sweet) scents; Class C: baked scents; Class D: unpleasant scents.

## Supplementary Materials

The following are available online, Table S1: information and sensory evaluations of collected white tea samples, Table S2: identified volatile compounds and their contents in white teas, Table S3: the detailed performance of GC–O analysis of Baihaoyinzhen, Table S4: the detailed performance of GC–O analysis of Baimudan, Table S5: the detailed performance of GC–O analysis of Shoumei, Figure S1: typical overlay plot of total ion map and GC–O time-intensity map.
