# Supplementary material for: Identification of Aroma Composition and Key Odorants Contributing to Aroma Characteristics of White Teas
Source: Molecules. 2020 Dec 21;25(24):6050. doi: 10.3390/molecules25246050 (PMC7767441; doi:10.3390/molecules25246050)
Supplement: Supplementary file 1 [file molecules-25-06050-s001.pdf]

Identification of Aroma Composition and Key Odorants Contributing to Aroma Characteristics of White Teas

Qin-Cao Chen [1,2,3,†], Yin Zhu [1,†], Han Yan [1], Mei Chen [1], Dong-Chao Xie [1], Meng-Qi Wang [1], De-Jiang Ni [3,*] and Zhi Lin [1,*]

[1] Tea Research Institute, Chinese Academy of Agricultural Sciences, No.9 Meiling South Road, West lake District, Hangzhou 310008, China; chenqincao@jxau.edu.cn (Q.-C.C.); zhuy_scu@tricaas.com (Y.Z.); yanhan@tricaas.com (H.Y.); chenxiaomei88520@163.com (M.C.); superxie@tricaas.com (D.-C.X.); lfwangmengqife@126.com (M.-Q.W.)

[2] College of Agriculture, Jiangxi Agricultural University, No. 1101 Zhimin Avenue, Qingshan Lake District, Nanchang 330045, China

[3] College of Horticulture and Forestry Science, Huazhong Agricultural University, No.1 Shizishan Street, Hongshan District, Wuhan 430070, China

* Correspondence: nidj@mail.hzau.edu.cn (D.-J.N.); linzhi@caas.cn (Z.L.)

† These authors contributed to this work equally.

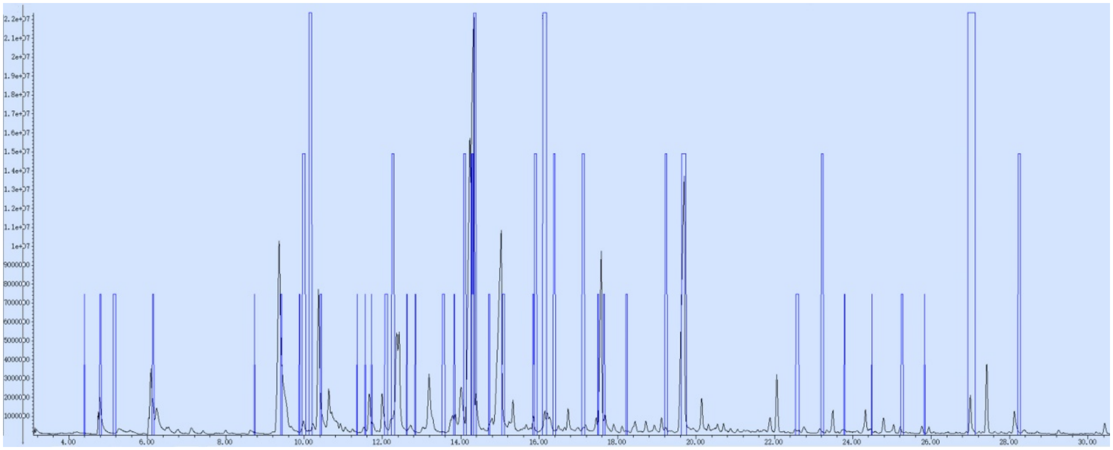

Figure S1. Typical overlay plot of total ion map and GC-O time-intensity map.

Table S1. Information and sensory evaluations of collected white tea samples.

NO.	Class	Sensory evaluation	Score
1	Baihaoyinzhen	Clean and sweet, have pekoe flavor, slight sulky odor	86
2	Baihaoyinzhen	Have pekoe flavor, cream-like, slight stale odor	84
3*	Baihaoyinzhen	Clean and sweet, lasting pekoe flavor, slight sulky odor	90
4	Baihaoyinzhen	Clean, sweet, high pekoe flavor	94
5	Baihaoyinzhen	Have pekoe flavor, slight fired aroma	84
6	Baimudan	Clean and sweet, high pekoe flavor, slight sulky odor	90
7	Baimudan	Have pekoe flavor, slight sulky aroma	84
8*	Baimudan	Lasting pekoe flavor, slight sulky odor	88
9	Baimudan	High pekoe flavor	94
10	Baimudan	Smoke-like, slight pekoe flavor, have sulky odor	78
11	Baimudan	Clean and sweet, have pekoe flavor	90
12	Shoumei	Slight pekoe flavor, have sulky odor	83
13	Shoumei	Pure and normal, slightly clean	86
14	Shoumei	Approach high odor, have sulky odor, slightly clean	85
15*	Shoumei	Normal, little fermentation odor, sweet	80
16	Shoumei	Pure and normal, slightly grassy	85
17	Shoumei	Slight harsh odor, have sulky odor	78

Note: * the tea samples were used for GC-O/MS analysis.

Table S2. Identified volatile compounds and their contents in white teas[1].

NO.	Compounds	CAS	1st DT	2nd DT	RI	Average peak area	BHYZ		BMD		SM		*P*-Value[3]	VIP
							Average ratio[2]	Range[2]	Average ratio[2]	Range[2]	Average ratio[2]	Range[2]		
	**Alkanes**													
1	Octane	111-65-9	5.2500	1.62	801	78518±32222	0.73±0.14	0.61~0.89	0.90±0.06	0.80~0.97	1.37±0.45	0.85~1.97	0.000	1.007
2	2,4-Dimethyl-heptane	2213-23-2	5.6667	1.62	822	19488±11298	0.81±0.39	0.52~1.48	0.85±0.38	0.61~1.52	1.30±0.72	0.65~2.45	0.034	0.864
3	Nonane	111-84-2	7.1667	1.68	897	28496±6986	0.95±0.17	0.79~1.20	0.94±0.17	0.83~1.23	1.11±0.18	0.89~1.30	0.052	0.815
4	4-Ethyl-octane	15869-86-0	8.3333	1.69	956	8982±4854	0.83±0.24	0.62~1.22	0.67±0.20	0.42~0.88	1.50±0.42	1.07~2.20	0.000	1.169
5	3-Methyl-nonane	5911-04-6	8.6667	1.69	972	62453±29885	0.81±0.16	0.70~1.10	0.74±0.20	0.49~0.92	1.44±0.31	1.20~1.89	0.000	1.090
6	2,2,4,6,6-Pentamethyl-heptane	13475-82-6	9.0833	1.68	993	260891±105173	1.01±0.37	0.56~1.59	0.73±0.23	0.39~1.02	1.27±0.27	0.90~1.55	0.002	1.196
7	Decane	124-18-5	9.2500	1.74	1002	2953769±672766	0.95±0.17	0.75~1.14	0.93±0.10	0.82~1.03	1.12±0.14	0.91~1.26	0.068	0.816
8	Pentyl-cyclopentane	3741-00-2	10.0833	1.77	1043	13451±5461	0.81±0.24	0.48~1.11	0.78±0.10	0.67~0.90	1.40±0.25	1.09~1.67	0.000	1.164
9	Indane	496-11-7	10.0833	2.52	1044	8564±3356	1.04±0.49	0.53~1.82	0.77±0.18	0.52~1.02	1.19±0.08	1.10~1.29	0.005	1.065
10	3-Methyl-undecane	1002-43-3	12.5833	1.77	1171	355209±110232	0.98±0.10	0.81~1.05	0.82±0.11	0.71~0.97	1.19±0.31	0.81~1.64	0.007	1.086
11	Dodecane	112-40-3	13.1667	1.78	1207	686940±165274	0.97±0.12	0.77~1.08	0.94±0.16	0.78~1.18	1.09±0.23	0.81~1.37	0.095	0.807
12	5-Ethyl-undecane	17453-94-0	13.9167	1.76	1245	21156±11401	1.11±0.44	0.65~1.68	0.88±0.13	0.71~1.00	1.02±0.38	0.54~1.47	0.593	0.694
13	Tridecane	629-50-5	14.9167	1.79	1302	145461±74731	0.95±0.19	0.78~1.26	0.7±0.070	0.60~0.79	1.35±0.40	0.85~1.78	0.001	1.075
14	3-Methyl-tridecane	6418-41-3	16.0833	1.80	1372	277594±104681	1.07±0.13	0.93~1.23	0.76±0.09	0.64~0.88	1.17±0.37	0.69~1.55	0.007	1.106
15	Tetradecane	629-59-4	16.5833	1.81	1402	334934±120679	1.02±0.21	0.76~1.32	0.81±0.07	0.72~0.88	1.18±0.29	0.80~1.49	0.018	0.974
16	2-Methyl-tridecane	1560-96-9	18.1667	1.75	1502	3995±2268	1.26±0.83	0.46~2.47	0.82±0.40	0.38~1.34	0.97±0.18	0.67~1.11	0.426	1.061
17	3-Methyl-pentadecane	2882-96-4	19.2500	1.83	1574	89351±36909	1.25±0.28	0.95~1.69	0.79±0.12	0.59~0.91	0.96±0.18	0.73~1.15	0.017	1.168
	**Alkenes**													
18	(E)-2-Pentene	646-04-8	2.5000	1.39	525	27539±17945	0.39±0.19	0.24~0.72	1.11±0.46	0.66~1.83	1.52±0.21	1.17~1.73	0.000	1.302
19	1,3-Pentadiene	504-60-9	2.5000	1.44	526	99041±56883	0.96±0.82	0.48~2.41	0.89±0.38	0.53~1.52	1.15±0.20	0.81~1.32	0.004	0.460
20	1-Octene	111-66-0	5.0833	1.67	791	8111±4078	0.77±0.13	0.59~0.88	0.86±0.08	0.76~0.98	1.37±0.43	0.91~1.84	0.001	0.807
21	(Z)-3-Octene	14919-01-8	5.3333	1.70	814	5837±3322	0.87±0.42	0.60~1.61	1.14±0.50	0.63~1.71	0.92±0.33	0.64~1.43	0.656	0.906
22	α-Pinene	80-56-8	8.0000	1.88	939	122452±213882	0.42±0.37	0.10~1.04	1.91±3.05	0.24~7.34	0.67±0.32	0.16~1.02	0.071	1.099
23	(E,E)-2,4-Nonadiene	56700-78-8	8.0833	2.05	943	58962±28060	0.70±0.33	0.41~1.21	0.99±0.48	0.46~1.69	1.32±0.33	0.75~1.55	0.001	1.008
24	3-Methylene-nonane	51655-64-2	9.0000	1.76	989	36793±15838	0.79±0.14	0.64~1.02	0.76±0.10	0.65~0.89	1.44±0.20	1.24~1.75	0.000	1.180
25	β-Myrcene	123-35-3	9.0833	1.97	993	785254±200748	1.07±0.27	0.61~1.31	1.09±0.14	0.88~1.25	0.83±0.12	0.71~1.00	0.006	0.946
26	α-Phellandrene	99-83-2	9.4167	2.03	1010	44910±15354	1.07±0.33	0.66~1.51	0.92±0.23	0.67~1.28	1.04±0.17	0.87~1.23	0.166	0.865
27	Limonene	138-86-3	9.9167	2.05	1035	437124±161651	0.86±0.27	0.55~1.25	1.14±0.54	0.78~2.10	1.00±0.10	0.87~1.13	0.061	1.032
28	3-Ethyl-2-methyl-1,3-hexadiene	61142-36-7	10.0000	2.54	1040	53485±18585	0.93±0.16	0.78~1.18	0.80±0.20	0.53~1.00	1.27±0.35	0.77~1.75	0.001	1.085
29	β-Ocimene	13877-91-3	10.2500	2.03	1052	276890±77092	1.06±0.31	0.56~1.35	1.10±0.12	0.92~1.23	0.84±0.11	0.70~1.01	0.012	0.928

30	(E)-4,8-Dimethylnona-1,3,7-triene	19945-61-0	11.5833	2.04	1119	30644±17287	0.80±0.09	0.69~0.90	0.73±0.16	0.54~0.96	1.44±0.68	0.83~2.59	0.000	0.986
31	1,3,8-p-Menthatriene	18368-95-1	11.7500	2.37	1128	71022±33565	0.75±0.39	0.45~1.40	0.81±0.23	0.58~1.07	1.43±0.24	1.18~1.77	0.000	1.062
32	2,6-Dimethyl-1,3,5,7-octatetraene	460-01-5	11.9167	2.21	1137	21036±8885	1.00±0.47	0.49~1.71	0.93±0.28	0.54~1.25	1.08±0.31	0.78~1.45	0.432	0.631
33	3,3-Dimethyl-1-hexene	74511-51-6	12.0000	1.79	1141	182227±72325	0.93±0.26	0.62~1.22	0.90±0.07	0.77~0.96	1.17±0.26	1.01~1.64	0.187	0.682
34	(4E,6Z)-allo-Ocimene	7216-56-0	12.1667	2.13	1150	34706±10834	1.07±0.25	0.63~1.27	1.00±0.08	0.88~1.10	0.94±0.16	0.77~1.19	0.743	0.577
35	α-Cubebene	17699-14-8	15.9167	2.07	1362	25708±14377	1.26±0.34	0.79~1.69	1.09±0.64	0.53~2.19	0.66±0.44	0.32~1.42	0.001	0.838
36	α-Copaene	3856-25-5	16.4167	2.13	1392	12882±9970	0.99±0.58	0.29~1.71	0.73±0.57	0.30~1.50	1.23±0.96	0.49~2.90	0.232	0.674
37	α-Cedrene*	469-61-4	17.0833	2.27	1439	400059±434514	0.83±0.65	0.12~1.66	0.88±1.21	0.13~2.99	1.29±1.45	0.14~3.81	0.433	0.626
38	β-Cedrene	546-28-1	17.2500	2.31	1445	90103±106118	0.85±0.69	0.10~1.75	0.81±1.21	0.10~2.95	1.34±1.65	0.10~4.21	0.360	0.609
39	cis-Thujopsene	470-40-6	17.4167	2.32	1455	29176±39358	0.63±0.57	0.05~1.43	0.79±0.97	0.08~2.46	1.45±2.14	0.03~5.22	0.658	0.687
40	cis-β-Farnesene	28973-97-9	17.5833	2.08	1465	10728±6323	1.03±0.42	0.51~1.56	0.88±0.42	0.50~1.42	1.08±0.76	0.59~2.41	0.698	0.597
41	α-Selinene	473-13-2	18.2500	2.34	1508	27525±33497	0.83±0.66	0.16~1.67	0.71±0.76	0.19~2.04	1.40±1.83	0.15~4.62	0.508	0.633
42	α-Alaskene	28400-12-6	18.5833	2.34	1530	29799±43392	0.67±0.67	0.09~1.72	0.63±0.98	0.07~2.36	1.64±2.25	0.10~5.56	0.137	0.732
43	Cadinadiene	29837-12-5	18.9167	2.36	1553	6338±3496	1.20±0.36	0.79~1.55	1.12±0.59	0.5~2.11	0.62±0.40	0.31~1.31	0.002	0.910
44	Caryophyllene oxide	1139-30-6	19.7500	2.61	1609	5256±4019	0.96±0.48	0.43~1.55	0.91±1.00	0.29~2.67	0.95±0.81	0.39~2.39	0.426	0.531
45	Neophytadiene	504-96-1	22.9167	1.94	1843	3433±1382	0.81±0.08	0.75~0.91	0.79±0.09	0.69~0.91	1.40±0.17	1.12~1.55	0.000	1.154
	**Aromatic hydrocarbons**													
46	Toluene	108-88-3	4.7500	1.99	769	706579±195838	0.88±0.20	0.74~1.22	0.95±0.22	0.68~1.22	1.18±0.17	1.03~1.45	0.005	0.738
47	1,3-Dimethyl-benzene	108-38-3	6.6667	2.12	873	173308±65003	0.83±0.23	0.51~1.16	0.74±0.13	0.52~0.86	1.42±0.16	1.18~1.61	0.000	1.343
48	o-Xylene	95-47-6	7.0833	2.22	898	73205±29276	0.81±0.18	0.58~1.04	0.71±0.13	0.49~0.80	1.48±0.16	1.27~1.71	0.000	1.411
49	Styrene	100-42-5	7.0833	2.32	894	307669±111520	0.70±0.15	0.56~0.87	0.92±0.25	0.62~1.29	1.38±0.16	1.17~1.58	0.000	1.180
50	Propyl-benzene	103-65-1	8.4167	2.19	960	18856±6076	0.80±0.05	0.76~0.87	0.89±0.11	0.77~1.06	1.31±0.15	1.19~1.57	0.000	1.051
51	1-Ethyl-4-methyl-benzene	622-96-8	8.5833	2.18	968	44980±17673	0.84±0.16	0.60~1.00	0.77±0.21	0.43~0.93	1.39±0.14	1.24~1.61	0.000	1.180
52	1-Ethyl-2-methyl-benzene	611-14-3	8.9167	2.25	985	13223±4878	0.88±0.24	0.60~1.23	0.79±0.12	0.62~0.89	1.34±0.10	1.26~1.51	0.000	1.129
53	Mesitylene	108-67-8	9.1667	2.29	998	63020±23341	0.78±0.20	0.56~1.07	0.83±0.16	0.65~0.98	1.39±0.02	1.36~1.40	0.000	1.175
54	β-Cymene	535-77-3	9.8333	2.18	1031	199712±89624	0.87±0.26	0.60~1.29	1.13±0.64	0.79~2.28	1.00±0.10	0.90~1.15	0.184	0.913
55	1,2,3-Trimethyl-benzene	526-73-8	9.8333	2.35	1031	58368±31300	0.61±0.38	0.25~1.20	0.89±0.23	0.62~1.15	1.51±0.11	1.41~1.68	0.000	1.116
56	2-p-Tolylpropene	1195-32-0	11.1667	2.38	1098	96371±38183	0.89±0.25	0.55~1.12	1.01±0.56	0.65~2.00	1.09±0.23	0.85~1.32	0.099	0.689
57	1,2,4,5-Tetramethyl-benzene	95-93-2	11.7500	2.18	1128	20301±10725	0.90±0.44	0.55~1.59	0.86±0.17	0.66~1.11	1.24±0.48	0.92~2.04	0.052	0.646
58	4-Methylindan	824-22-6	12.1667	2.51	1150	6954±2968	0.79±0.32	0.44~1.29	0.84±0.22	0.55~1.04	1.37±0.22	1.15~1.64	0.000	1.044
59	Naphthalene*	91-20-3	13.0833	2.97	1198	418668±163229	0.91±0.35	0.56~1.48	1.06±0.45	0.61~1.64	1.06±0.28	0.88~1.49	0.172	0.791
60	1-Methyl-naphthalene	90-12-0	15.0833	2.93	1313	47892±13597	0.90±0.19	0.65~1.16	0.92±0.19	0.60~1.09	1.18±0.22	0.90~1.46	0.014	0.811
61	Biphenyl	92-52-4	16.5000	3.01	1398	6691±2301	1.09±0.36	0.84~1.73	0.99±0.25	0.73~1.37	0.91±0.20	0.71~1.20	0.528	0.576
62	1,3-Dimethyl-naphthalene	575-41-7	16.9167	2.89	1423	5582±1829	0.92±0.18	0.70~1.16	1.04±0.37	0.64~1.63	1.05±0.19	0.80~1.24	0.458	0.616
63	1,4-Dimethyl-naphthalene	571-58-4	17.1667	2.98	1440	10965±4631	0.89±0.26	0.56~1.15	1.10±0.47	0.54~1.82	1.01±0.28	0.68~1.30	0.536	0.716
64	α-Curcumene	644-30-4	18.0833	2.28	1497	27659±20890	0.91±0.53	0.39~1.76	0.93±0.59	0.56~1.98	1.16±0.99	0.40~2.90	0.699	0.557
65	Cuparene	16982-00-6	18.5833	2.53	1531	33989±39880	0.95±0.81	0.09~2.06	0.68±0.89	0.16~2.26	1.38±1.71	0.08~4.33	0.439	0.652
66	cis-Calamenene	72937-55-4	18.7500	2.48	1542	69606±31012	1.27±0.34	0.80~1.76	0.94±0.31	0.53~1.24	0.79±0.33	0.39~1.17	0.024	0.901

67	α-Calacorene	21391-99-1	19.0833	2.59	1564	14571±5302	1.15±0.38	0.86~1.79	0.96±0.18	0.75~1.20	0.89±0.23	0.63~1.26	0.291	0.800
	**Alcohols**													
68	1-Penten-3-ol	616-25-1	3.5833	1.77	685	519279±185257	1.24±0.29	0.86~1.68	1.07±0.21	0.87~1.38	0.69±0.15	0.53~0.89	0.000	0.991
69	2-Methyl-1-butanol	137-32-6	4.2500	1.86	735	58524±23444	1.05±0.39	0.64~1.59	0.85±0.17	0.73~1.13	1.10±0.51	0.31~1.71	0.125	0.725
70	1-Pentanol	71-41-0	4.6667	1.95	763	190596±79548	1.23±0.37	0.61~1.57	1.16±0.25	0.84~1.42	0.62±0.19	0.44~0.93	0.000	1.106
71	2-Methyl-3-pentanol	565-67-3	4.7500	1.88	769	25765±12961	1.40±0.37	0.96~1.87	1.16±0.05	1.11~1.21	0.44±0.12	0.30~0.57	0.000	1.225
72	(Z)-2-Penten-1-ol	1576-95-0	4.7500	2.02	769	171370±69755	1.07±0.44	0.39~1.47	1.18±0.29	0.77~1.46	0.75±0.28	0.44~1.17	0.011	1.032
73	(Z)-3-Hexen-1-ol	544-12-7	6.3333	2.17	856	1315779±393193	0.96±0.22	0.79~1.33	0.94±0.16	0.78~1.19	1.11±0.32	0.74~1.61	0.220	0.772
74	1-Hexanol*	111-27-3	6.5833	2.06	868	357833±124725	0.92±0.25	0.67~1.28	0.92±0.15	0.68~1.06	1.16±0.45	0.51~1.71	0.141	0.531
75	1-Heptanol	111-70-6	8.6667	2.10	973	64394±31328	0.65±0.24	0.49~1.07	0.87±0.24	0.56~1.22	1.48±0.38	0.94~1.98	0.000	1.103
76	1-Octen-3-ol*	3391-86-4	8.8333	2.09	981	628533±182436	1.12±0.16	0.90~1.29	0.89±0.27	0.64~1.30	0.98±0.20	0.68~1.17	0.081	0.846
77	2-Ethyl-1-hexanol	104-76-7	9.9167	2.07	1035	26343±28997	1.03±0.70	0.35~2.20	0.50±0.29	0.11~0.82	1.47±1.47	0.21~3.97	0.063	0.867
78	Benzyl alcohol	100-51-6	10.0000	2.93	1040	2381805±847000	1.32±0.18	1.12~1.55	1.02±0.16	0.79~1.23	0.66±0.10	0.58~0.83	0.000	1.283
79	1-Octanol	111-87-5	10.7500	2.12	1077	121448±56633	0.66±0.23	0.42~1.02	0.91±0.15	0.74~1.12	1.43±0.42	0.77~1.80	0.000	1.109
80	(E)-2-Octen-1-ol	18409-17-1	10.7500	2.18	1077	50001±24788	1.11±0.23	0.81~1.40	0.92±0.32	0.37~1.18	0.98±0.34	0.70~1.53	0.348	0.563
81	cis-Linalool Oxide (furanoid)	5989-33-3	10.8333	2.17	1081	178091±113291	0.45±0.05	0.39~0.52	0.75±0.11	0.60~0.89	1.80±0.21	1.54~2.10	0.000	1.375
82	trans-Linalool oxide (furanoid)*	34995-77-2	11.0833	2.24	1094	303101±188191	0.48±0.16	0.23~0.66	0.78±0.16	0.60~1.00	1.74±0.20	1.46~1.96	0.000	1.320
83	Linalool*	78-70-6	11.3333	2.17	1106	6600625±816965	0.99±0.10	0.82~1.07	0.99±0.05	0.91~1.04	1.02±0.12	0.91~1.23	0.939	0.615
84	Phenylethyl Alcohol	60-12-8	11.5833	2.97	1120	6642753±2886122	1.46±0.24	1.22~1.74	0.94±0.20	0.62~1.14	0.60±0.08	0.54~0.71	0.000	1.415
85	(Z)-3-Nonen-1-ol*	10340-23-5	12.3333	2.22	1159	87496±40175	1.10±0.14	0.93~1.27	1.09±0.28	0.77~1.47	0.81±0.42	0.48~1.50	0.011	0.628
86	1-Nonanol*	143-08-8	12.6667	2.13	1176	163082±76200	0.65±0.32	0.27~1.12	1.08±0.24	0.74~1.31	1.27±0.39	0.60~1.60	0.001	1.140
87	trans-linalool oxide (pyranoid)	39028-58-5	12.7500	2.42	1180	94059±44985	0.60±0.13	0.41~0.74	0.89±0.24	0.69~1.28	1.51±0.13	1.40~1.69	0.000	1.268
88	a,a,4-Trimethylbenzyl alcohol	1197-01-9	13.0000	2.65	1194	11563±14287	0.87±0.80	0.37~2.07	1.18±2.02	0.20~4.80	0.78±0.23	0.53~1.04	0.039	0.728
89	α-Terpineol	98-55-5	13.0833	2.44	1198	144842±91795	0.83±0.49	0.37~1.64	0.83±0.64	0.41~1.96	1.34±0.69	0.69~2.39	0.014	0.781
90	γ-Isogeraniol	13066-51-8	13.5833	2.29	1226	10095±3455	1.01±0.30	0.55~1.37	0.90±0.19	0.75~1.21	1.09±0.21	0.79~1.37	0.465	0.708
91	Nerol	106-25-2	13.7500	2.30	1236	115721±41866	1.00±0.22	0.72~1.34	0.96±0.06	0.89~1.01	1.03±0.31	0.72~1.41	0.705	0.626
92	Geraniol	106-24-1	14.1667	2.37	1259	4039293±1675592	1.16±0.33	0.65~1.55	1.26±0.32	0.95~1.71	0.58±0.08	0.52~0.72	0.000	1.275
93	Nerolidol	142-50-7	19.2500	2.24	1575	40329±15952	1.17±0.22	0.84~1.42	0.92±0.34	0.61~1.47	0.92±0.27	0.59~1.20	0.105	0.772
94	Cedrol	77-53-2	20.0833	2.67	1633	190325±193607	1.16±1.02	0.08~2.6	0.48±0.46	0.06~1.17	1.35±1.37	0.07~3.64	0.058	0.943
	**Aldehydes**													
95	2-Methyl-propanal	78-84-2	2.6667	1.55	566	333718±158722	0.95±0.35	0.71~1.56	0.92±0.39	0.56~1.59	1.13±0.39	0.67~1.64	0.663	0.387
96	Isobutenal	78-85-3	2.7500	1.58	586	10253±6815	0.77±0.32	0.50~1.31	0.87±0.50	0.54~1.75	1.36±0.34	1.03~1.80	0.025	0.630
97	Butanal	123-72-8	2.8333	1.64	603	888969±427628	1.37±0.28	1.09~1.71	1.01±0.24	0.65~1.26	0.58±0.26	0.23~0.83	0.000	1.087
98	3-Methyl-butanal	590-86-3	3.3333	1.74	658	363692±100638	1.08±0.11	1.02~1.27	0.83±0.15	0.64~1.06	1.09±0.36	0.66~1.61	0.010	1.109
99	(E)-2-Butenal	123-73-9	3.3333	1.88	658	46678±20028	1.27±0.32	0.90~1.71	0.78±0.23	0.54~1.08	0.95±0.38	0.65~1.57	0.005	1.148
100	2-Methyl-butanal	96-17-3	3.4167	1.76	667	1477185±681540	0.93±0.28	0.73~1.41	0.86±0.23	0.71~1.25	1.21±0.56	0.48~1.74	0.261	0.623
101	Pentanal	110-62-3	3.7500	1.87	702	316385±90106	1.20±0.10	1.06~1.31	0.90±0.20	0.61~1.10	0.09±0.30	0.69~1.44	0.002	1.085
102	trans-2-Methyl-2-butenal	497-03-0	4.3333	2.10	747	117964±81280	1.40±0.69	0.46~2.12	1.13±0.68	0.46~2.06	0.47±0.35	0.18~1.05	0.000	0.953

103	(E)-2-Pentenal	1576-87-0	4.5833	2.14	758	135788±57635	0.96±0.04	0.91~1.00	0.84±0.24	0.54~1.08	1.21±0.60	0.79~2.22	0.144	0.692
104	Hexanal	66-25-1	5.2500	2.10	802	2577877±595243	1.18±0.05	1.11~1.24	0.89±0.15	0.66~1.04	0.93±0.25	0.69~1.34	0.001	1.267
105	2-Ethyl-trans-2-butenal	63883-69-2	5.6667	2.19	823	29889±24637	1.61±1.03	0.48~2.87	0.99±0.58	0.48~1.96	0.39±0.17	0.24~0.62	0.000	1.045
106	2-Methyl-2-pentenal	623-36-9	5.9167	2.19	835	307754±278322	1.30±1.15	0.18~3.23	1.34±0.81	0.29~2.17	0.36±0.36	0.06~0.89	0.000	0.958
107	Furfural	98-01-1	5.9167	2.74	836	59752±52981	1.61±1.38	0.57~3.75	0.66±0.26	0.41~0.97	0.73±0.39	0.35~1.21	0.035	1.091
108	(Z)-2-Hexenal	16635-54-4	6.1667	2.23	848	42423±16490	0.84±0.14	0.61~0.98	0.82±0.18	0.63~1.08	1.33±0.28	1.03~1.78	0.000	1.115
109	(E)-2-Hexenal	6728-26-3	6.2500	2.33	852	5905484±1429938	0.89±0.11	0.78~1.02	0.84±0.09	0.78~0.99	1.27±0.12	1.14~1.39	0.000	1.360
110	Heptanal	111-71-7	7.2500	2.14	902	312423±131880	0.80±0.10	0.71~0.96	0.68±0.09	0.56~0.78	1.51±0.21	1.26~1.74	0.000	1.452
111	(Z)-4-Heptenal	6728-31-0	7.2500	2.25	902	14751±7273	0.80±0.17	0.58~1.04	0.65±0.19	0.40~0.87	1.48±0.33	1.07~1.82	0.000	1.325
112	(E,E)-2,4-Hexadienal	142-83-6	7.4167	2.58	915	113204±40319	0.85±0.09	0.72~0.97	0.86±0.22	0.63~1.19	1.29±0.36	0.96~1.78	0.002	1.002
113	(Z)-2-Heptenal	57266-86-1	8.4167	2.32	960	83371±43396	1.10±0.36	0.68~1.39	0.68±0.27	0.35~1.01	1.22±0.51	0.78~1.88	0.003	1.058
114	Benzaldehyde	100-52-7	8.5000	2.94	965	13696325±2886497	1.14±0.19	0.84~1.37	1.04±0.15	0.85~1.19	0.82±0.08	0.71~0.91	0.000	1.027
115	2-ethyl-2-Hexenal	645-62-5	9.1667	2.24	998	18562±11728	1.33±0.70	0.47~2.27	1.11±0.57	0.30~1.87	0.56±0.30	0.28~1.02	0.002	0.855
116	2,4-Heptadienal isomer1*	5910-85-0	9.2500	2.49	1002	265051±204509	0.55±0.11	0.40~0.70	0.65±0.22	0.42~0.96	1.81±0.89	1.24~3.33	0.000	1.135
117	Octanal*	124-13-0	9.3333	2.19	1006	368927±184613	0.69±0.11	0.55~0.79	0.76±0.16	0.53~0.93	1.54±0.41	1.03~2.10	0.000	1.210
118	2,4-Heptadienal isomer2*	5910-85-0	9.5000	2.56	1015	806708±683441	0.56±0.10	0.42~0.68	0.69±0.33	0.37~1.18	1.74±1.16	1.10~3.82	0.000	0.956
119	2-hydroxy-Benzaldehyde	90-02-8	10.2500	2.91	1052	9628±2519	0.86±0.09	0.70~0.93	0.96±0.23	0.74~1.22	1.18±0.20	0.89~1.38	0.006	1.137
120	Benzeneacetaldehyde*	122-78-1	10.2500	2.96	1053	1795094±1208323	0.48±0.08	0.36~0.57	0.88±0.20	0.66~1.17	1.65±0.81	0.90~3.01	0.000	1.106
121	(E)-2-Octenal*	2548-87-0	10.5000	2.32	1064	46224±32412	0.74±0.10	0.59~0.84	0.62±0.17	0.46~0.86	1.64±0.92	1.03~3.12	0.000	1.084
122	4-methyl-Benzaldehyde	104-87-0	10.7500	2.88	1077	18794±5705	1.09±0.22	0.78~1.33	0.89±0.28	0.64~1.34	1.02±0.20	0.88~1.37	0.273	0.916
123	(E,E)-2,4-Octadienal	30361-28-5	11.4167	2.56	1110	25886±13674	1.13±0.66	0.68~2.27	0.85±0.35	0.59~1.46	1.01±0.33	0.73~1.48	0.149	0.680
124	Nonanal*	124-19-6	11.4167	2.19	1111	1149050±556076	0.76±0.10	0.62~0.90	0.74±0.34	0.18~1.09	1.48±0.46	1.03~2.04	0.000	1.140
125	2-Methylenehexanal	1070-66-2	11.9167	2.26	1137	90940±70385	1.27±0.75	0.48~2.44	1.32±0.79	0.58~2.35	0.41±0.30	0.10~0.86	0.000	1.057
126	(E,Z)-2,6-Nonadienal	557-48-2	12.3333	2.43	1159	121680±46997	0.98±0.27	0.63~1.26	1.06±0.28	0.69~1.39	0.96±0.29	0.60~1.36	0.959	0.558
127	(E)-2-Nonenal*	18829-56-6	12.4167	2.31	1163	42336±19292	1.01±0.19	0.78~1.22	0.98±0.36	0.60~1.54	1.01±0.28	0.64~1.30	0.416	0.388
128	3-Ethyl-benzaldehyde	34246-54-3	12.5833	2.82	1172	13784±5640	1.33±0.31	1.05~1.82	1.02±0.14	0.86~1.20	0.66±0.20	0.42~0.82	0.000	1.150
129	Decanal	112-31-2	13.2500	2.19	1207	157723±67676	0.77±0.21	0.53~1.07	0.95±0.16	0.78~1.16	1.29±0.52	0.68~2.09	0.006	0.932
130	Safranal	116-26-7	13.3333	2.66	1212	22655±10719	0.83±0.23	0.53~1.09	0.67±0.24	0.42~1.05	1.50±0.34	1.15~2.01	0.000	1.285
131	(E,E)-2,4-Nonadienal	5910-87-2	13.5000	2.47	1221	35995±18167	0.77±0.26	0.51~1.17	0.80±0.24	0.54~1.17	1.43±0.58	0.78~2.14	0.000	1.012
132	β-Cyclocitral	432-25-7	13.6667	2.60	1231	72271±40160	0.58±0.09	0.47~0.68	0.69±0.13	0.50~0.85	1.73±0.12	1.62~1.92	0.000	1.437
133	Neral	106-26-3	14.0000	2.46	1250	160966±107640	0.99±0.29	0.53~1.31	0.92±0.05	0.87~0.97	1.10±0.50	0.61~1.73	0.124	0.431
134	(Z)-2-Decenal	2497-25-8	14.3333	2.12	1269	30015±15503	0.74±0.16	0.55~0.97	0.80±0.12	0.70~1.00	1.46±0.62	1.00~2.56	0.000	0.997
135	β-Cyclohomocitral	472-66-2	14.3333	2.50	1269	7078±4323	0.50±0.18	0.26~0.69	0.72±0.22	0.38~0.95	1.74±0.24	1.52~2.14	0.000	1.335
136	Geranial	141-27-5	14.5000	2.48	1279	367856±136240	1.14±0.35	0.59~1.51	1.14±0.08	1.02~1.24	0.72±0.17	0.55~0.96	0.001	1.030
137	α-Ethylidene-phenylacetaldehyde	4411-89-6	14.5833	3.11	1284	16592±6850	0.99±0.43	0.67~1.50	1.07±0.23	0.88~1.47	0.94±0.49	0.48~1.72	0.478	0.710
138	(E,E)-2,4-Decadienal	25152-84-5	14.9167	2.39	1302	6446±4768	0.58±0.15	0.41~0.75	0.62±0.22	0.41~0.93	1.80±0.74	1.06~2.98	0.000	1.200
139	2-Butyl-2-octenal	13019-16-4	16.2500	2.18	1382	89102±59003	1.26±0.62	0.74~2.27	1.31±0.50	0.68~1.89	0.43±0.22	0.23~0.74	0.000	1.158
	**Ketones**													

140	2-Pentanone	107-87-9	3.6667	1.84	694	181824±76401	1.20±0.30	0.78~1.57	0.94±0.29	0.71~1.38	0.86±0.16	0.61~1.07	0.013	0.738
141	1-Penten-3-one	1629-58-9	3.6667	1.88	694	283976±109580	0.85±0.12	0.68~1.00	0.76±0.10	0.62~0.90	1.38±0.35	1.14~2.00	0.000	1.214
142	(E)-3-Penten-2-one	3102-33-8	4.3333	2.12	741	65161±23686	1.25±0.23	0.98~1.61	1.03±0.37	0.68~1.46	0.72±0.14	0.48~0.82	0.000	1.046
143	2-Methyl-3-pentanone	565-69-5	4.5000	1.91	752	73555±39910	1.41±0.54	0.63~1.95	1.14±0.26	0.94~1.57	0.46±0.10	0.28~0.56	0.000	1.135
144	4-Methyl-2,3-pentanedione	7493-58-5	5.0000	2.02	786	34224±12921	1.00±0.22	0.79~1.28	0.89±0.16	0.66~1.11	1.03±0.37	0.66~1.44	0.600	0.633
145	2-Hexanone	591-78-6	5.0833	2.04	791	70361±35810	1.26±0.48	0.61~1.77	1.13±0.20	0.84~1.36	0.61±0.19	0.34~0.85	0.000	0.939
146	3-Methyl-2-hexanone	2550-21-2	6.0000	2.02	839	95572±31802	1.22±0.26	0.97~1.65	1.04±0.15	0.88~1.24	0.74±0.19	0.55~0.99	0.000	1.006
147	2-Heptanone	110-43-0	7.0000	2.15	889	875390±291095	1.24±0.35	0.75~1.63	0.99±0.25	0.81~1.41	0.77±0.15	0.62~0.96	0.001	0.997
148	1-(2-Furanyl)-ethanone	1192-62-7	7.5000	2.76	915	15446±7802	1.37±0.31	0.82~1.54	0.81±0.14	0.65~1.02	0.82±0.64	0.37~1.95	0.001	1.189
149	Butyrolactone	96-48-0	7.5000	3.68	920	22489±13774	1.52±0.61	0.97~2.55	0.66±0.22	0.35~0.91	0.82±0.30	0.50~1.28	0.000	1.435
150	6-Methyl-2-heptanone	928-68-7	8.3333	2.12	956	75081±20613	0.92±0.14	0.75~1.03	0.89±0.14	0.72~1.08	1.18±0.24	0.83~1.42	0.023	0.885
151	2-Methyl-1-hepten-6-one	10408-15-8	8.5833	2.30	973	6539±3575	0.68±0.30	0.39~1.12	0.81±0.40	0.39~1.48	1.51±0.36	0.94~1.83	0.000	1.042
152	1-Octen-3-one	4312-99-6	8.8333	2.24	981	36097±17043	1.32±0.28	0.96~1.62	0.73±0.28	0.41~1.07	1.01±0.25	0.82~1.31	0.001	1.270
153	2,3-Octanedione	585-25-1	8.9167	2.17	985	70453±22891	1.10±0.29	0.95~1.62	0.84±0.17	0.73~1.14	1.05±0.23	0.75~1.28	0.032	0.948
154	6-Methyl-5-hepten-2-one	110-93-0	9.0000	2.30	989	895560±234851	1.13±0.22	0.83~1.41	0.86±0.20	0.66~1.20	1.00±0.13	0.83~1.14	0.014	1.092
155	2-Octanone	111-13-7	9.0833	2.19	994	106260±30165	1.13±0.17	0.99~1.38	1.00±0.28	0.75~1.46	0.86±0.19	0.54~1.01	0.031	0.790
156	1,4-Cyclohex-2-enedione	4505-38-8	9.9167	3.17	1036	6369±4455	1.87±0.14	1.66~2.00	0.72±0.31	0.45~1.11	0.42±0.08	0.36~0.56	0.000	1.663
157	3-Octen-2-one	1669-44-9	10.0833	2.33	1044	409162±141126	1.10±0.32	0.78~1.63	1.02±0.40	0.62~1.51	0.88±0.20	0.55~1.06	0.266	0.750
158	γ-Hexyl lactone	695-06-7	10.4167	3.26	1061	43195±25031	1.07±0.42	0.67~1.77	0.72±0.14	0.53~0.92	1.21±0.79	0.54~2.57	0.049	0.916
159	3-Methyl-2-cyclohexen-1-one	1193-18-6	10.5833	2.44	1069	65642±36424	0.63±0.15	0.45~0.81	0.68±0.12	0.50~0.80	1.69±0.22	1.42~1.93	0.000	1.365
160	3,5-Octadien-2-one isomer 1	38284-27-4	10.7500	2.49	1077	1298892±663496	1.17±0.48	0.50~1.79	1.10±0.63	0.53~2.03	0.73±0.25	0.39~1.08	0.045	0.839
161	3-Nonanone	925-78-0	11.0000	2.17	1089	37987±12335	0.88±0.20	0.63~1.18	0.90±0.21	0.63~1.18	1.22±0.16	1.01~1.44	0.007	0.921
162	3,5-Octadien-2-one isomer 2*	38284-27-4	11.1667	2.53	1098	1162294±611600	1.43±0.47	0.88~2.00	1.06±0.42	0.60~1.64	0.52±0.13	0.31~0.64	0.000	1.134
163	6-Methyl-3,5-heptadiene-2-one	1604-28-0	11.4167	2.57	1111	58196±18246	1.08±0.29	0.79~1.44	0.95±0.21	0.82~1.32	0.97±0.28	0.76~1.45	0.588	0.836
164	3-Nonen-2-one	14309-57-0	12.0833	2.30	1146	79060±24423	1.12±0.23	0.97~1.54	0.97±0.22	0.78~1.24	0.90±0.22	0.65~1.23	0.138	0.842
165	5-Ethyl-6-methyl-3-hepten-2-one	57283-79-1	12.1667	2.23	1150	512340±144312	1.08±0.19	0.87~1.29	1.07±0.14	0.94~1.23	0.86±0.21	0.52~1.08	0.061	0.912
166	6-Undecanone	927-49-1	14.4167	2.14	1278	19566±7709	0.97±0.26	0.63~1.33	1.20±0.23	0.91~1.41	0.82±0.22	0.47~1.01	0.040	1.224
167	2-Butyl-cyclohexanone	1126-18-7	14.4167	2.65	1274	20257±8353	1.27±0.39	0.97~1.93	1.02±0.08	0.91~1.11	0.71±0.16	0.49~0.88	0.001	1.019
168	3-Undecanone	2216-87-7	14.7500	2.16	1293	95870±33577	0.76±0.14	0.58~0.92	0.94±0.19	0.70~1.23	1.31±0.24	1.08~1.68	0.000	1.090
169	2-Undecanone	112-12-9	14.8333	2.17	1297	26041±6821	1.02±0.26	0.76~1.42	1.01±0.15	0.76~1.19	1.00±0.08	0.93~1.14	0.990	0.864
170	γ-Nonalactone*	104-61-0	16.0833	2.97	1373	89197±42248	1.45±0.49	0.97~2.20	0.83±0.13	0.71~1.01	0.72±0.17	0.49~0.94	0.000	1.400
171	cis-Jasmone	488-10-8	16.7500	2.82	1414	46513±29432	1.39±0.50	0.56~1.85	0.81±0.54	0.23~1.63	0.80±0.71	0.15~1.63	0.015	0.964
172	α-Ionone*	127-41-3	17.1667	2.47	1440	33384±12782	0.74±0.09	0.64~0.85	0.87±0.18	0.61~1.10	1.38±0.30	1.12~1.75	0.000	1.123
173	trans-Geranylacetone	3796-70-1	17.5000	2.35	1460	238633±80712	0.76±0.16	0.59~0.94	0.94±0.11	0.84~1.06	1.30±0.19	1.12~1.61	0.000	1.107
174	Coumarin	91-64-5	17.5000	3.92	1462	6855±2713	0.77±0.20	0.61~1.11	1.07±0.27	0.82~1.50	1.16±0.34	0.86~1.69	0.016	1.110
175	3-Tridecanone	1534-26-5	18.0833	2.15	1497	28018±6772	0.95±0.14	0.77~1.13	0.98±0.15	0.83~1.19	1.07±0.15	0.88~1.29	0.310	0.787
176	trans-β-Ionone*	79-77-6	18.1667	2.54	1503	113094±43452	0.76±0.07	0.68~0.84	0.89±0.20	0.63~1.15	1.35±0.36	1.07~1.97	0.000	1.043
177	3-Pentadecanone	18787-66-1	21.0000	2.17	1698	4247±1044	1.06±0.13	0.91~1.23	1.02±0.14	0.88~1.17	0.93±0.09	0.81~1.02	0.394	0.655

178	(1,2)-Bis-nor-phytone	502-69-2	23.0000	2.12	1850	37917±11566	1.19±0.09	1.11~1.32	1.00±0.06	0.93~1.07	0.81±0.13	0.63~0.96	0.013	0.966
	**Esters**													
179	Pentyl formate	638-49-3	5.7500	1.97	827	21045±7984	1.01±0.37	0.72~1.45	1.08±0.33	0.57~1.38	0.88±0.28	0.50~1.20	0.365	1.019
180	cis-3-Hexenyl formate	33467-73-1	7.5833	2.13	918	24947±9226	0.88±0.24	0.61~1.21	1.00±0.44	0.61~1.54	1.12±0.25	0.81~1.47	0.204	0.755
181	(Z)-Methyl hex-3-enoate	13894-62-7	7.8333	2.20	931	25043±19580	1.21±0.50	0.65~1.79	0.66±0.20	0.37~0.84	1.13±1.20	0.30~3.24	0.012	0.849
182	Methyl 2-hexenoate	2396-77-2	8.5833	2.27	969	14013±5337	0.92±0.21	0.7~1.160	0.92±0.24	0.63~1.26	1.16±0.50	0.48~1.69	0.290	0.678
183	cis-3-Hexenyl Acetate	3681-71-8	9.4176	2.19	1010	90078±38532	0.87±0.31	0.64~1.32	0.99±0.34	0.71~1.53	1.05±0.59	0.26~1.87	0.269	0.554
184	Methyl heptanoate	106-73-0	9.7500	2.11	1027	17218±8830	0.95±0.55	0.55~1.93	0.84±0.16	0.62~1.01	1.21±0.64	0.70~2.27	0.169	0.681
185	Benzyl formate	104-57-4	10.9167	2.80	1086	8396±3503	1.14±0.51	0.68~1.80	0.97±0.34	0.64~1.46	0.89±0.22	0.67~1.20	0.577	0.839
186	Methyl benzoate	93-58-3	11.2500	2.78	1102	50812±15498	1.06±0.13	0.83~1.17	0.90±0.16	0.77~1.09	1.03±0.32	0.69~1.50	0.259	0.852
187	Methyl octanoate	111-11-5	11.7500	2.12	1128	37278±19146	0.86±0.53	0.45~1.78	0.98±0.22	0.70~1.20	1.16±0.62	0.61~2.22	0.169	0.702
188	Benzyl acetate	140-11-4	12.5833	2.78	1172	26845±11497	1.44±0.34	1.11~1.90	0.83±0.15	0.58~0.98	0.74±0.07	0.67~0.85	0.000	1.473
189	2-Phenylethyl formate	104-62-1	12.8333	2.81	1185	28164±13196	1.39±0.44	0.99~2.12	0.88±0.24	0.62~1.26	0.73±0.27	0.55~1.20	0.000	1.203
190	cis-3-Hexenyl butyrate*	16491-36-4	12.9167	2.17	1189	23463±23413	0.50±0.14	0.37~0.67	0.94±0.61	0.52~1.99	1.56±1.50	0.38~4.14	0.002	0.770
191	Hexyl butanoate	2639-63-6	13.0000	2.10	1193	3874±4360	0.36±0.17	0.20~0.58	0.57±0.24	0.27~0.92	1.96±1.48	0.44~4.39	0.000	1.005
192	(Z)-2-Hexenyl butanoate	56922-77-1	13.0000	2.19	1193	48453±17433	0.82±0.22	0.54~1.05	1.01±0.27	0.69~1.41	1.17±0.29	0.84~1.61	0.029	0.989
193	Methyl salicylate	119-36-8	13.1667	2.80	1207	2864652±1252426	0.77±0.23	0.53~1.06	1.18±0.46	0.73~1.87	1.05±0.41	0.60~1.70	0.055	1.179
194	Methyl nonanoate	1731-84-6	13.5833	2.13	1226	26004±14930	0.62±0.41	0.28~1.30	0.88±0.37	0.53~1.45	1.38±0.62	0.88~2.44	0.003	0.891
195	cis-3-Hexenyl isovalerate*	35154-45-1	13.8333	2.12	1240	53119±45974	0.41±0.07	0.31~0.48	0.79±0.19	0.50~1.03	1.81±1.10	0.93~3.67	0.000	1.065
196	Hexyl 2-methylbutanoate	10032-15-2	13.9167	2.05	1245	4610±4078	0.19±0.01	0.17~0.20	0.30±0.07	0.24~0.40	1.75±0.53	0.83~2.21	0.000	1.188
197	Isopentyl hexanoate	2198-61-0	14.0833	2.07	1254	3735±2012	0.69±0.23	0.53~1.10	0.81±0.29	0.47~1.26	1.47±0.52	0.60~1.85	0.001	1.081
198	2-Phenylethyl acetate	103-45-7	14.2500	2.76	1265	51529±26411	1.59±0.32	1.15~1.94	0.75±0.15	0.55~0.91	0.66±0.17	0.48~0.91	0.000	1.692
199	Pentyl hexanoate	540-07-8	14.7500	2.11	1293	3065±1633	0.73±0.17	0.56~1.00	1.17±0.39	0.80~1.71	1.08±0.71	0.43~2.28	0.023	1.077
200	Bornyl acetate	76-49-3	14.8333	2.34	1298	8308±9950	0.61±0.58	0.07~1.29	0.94±1.70	0.05~3.97	1.21±1.14	0.07~3.12	0.139	0.597
201	Methyl geraniate	2349-14-6	15.3333	2.34	1332	103456±47250	1.08±0.06	0.99~1.15	0.93±0.28	0.66~1.38	0.99±0.63	0.54~2.09	0.256	0.680
202	Hexyl tiglate	16930-96-4	15.5000	2.22	1337	2978±1253	1.21±0.44	0.86~1.97	0.95±0.22	0.69~1.27	0.84±0.21	0.56~1.04	0.142	0.895
203	2-Phenethyl propionate	122-70-3	15.9167	2.71	1363	4807±2469	1.49±0.46	1.08~2.17	0.96±0.16	0.85~1.23	0.56±0.16	0.42~0.83	0.000	1.313
204	(Z)-3-Hex-1-enyl benzoate	25152-85-6	19.4167	2.70	1586	8835±10975	0.58±0.13	0.42~0.77	0.66±0.25	0.38~1.04	1.76±2.04	0.57~5.37	0.008	0.693
205	E-2-Hexenyl benzoate	76841-70-8	19.5833	2.71	1598	3814±2844	0.84±0.18	0.63~1.08	0.65±0.19	0.40~0.84	1.42±1.13	0.67~3.42	0.023	0.921
206	2-Phenethyl hexanoate	6290-37-5	20.4167	2.64	1656	4266±1381	1.26±0.20	1.13~1.61	0.93±0.07	0.86~1.01	0.80±0.08	0.71~0.88	0.001	1.248
207	Diisobutyl phthalate	84-69-5	23.2500	2.93	1884	75018±133149	1.24±0.81	0.29~2.53	0.67±0.13	0.53~0.88	1.10±1.64	0.23~4.02	0.236	0.489
	**Acids**													
208	Hexanoic acid	142-62-1	8.7500	2.23	977	640143±448482	1.63±0.69	1.00~2.79	0.96±0.24	0.64~1.29	0.38±0.06	0.30~0.44	0.000	1.228
209	Octanoic acid	124-07-2	12.5000	2.18	1167	50035±34877	1.60±0.63	1.09~2.59	0.83±0.32	0.51~1.26	0.43±0.46	0.10~1.22	0.000	1.313
210	Nonanoic acid	112-05-0	14.2500	2.20	1269	87805±45998	1.36±0.47	0.99~1.90	0.82±0.23	0.56~1.09	0.82±0.49	0.24~1.42	0.006	1.079
	**Oxygen heterocyclic compounds**													
211	2-Methyl-furan	534-22-5	2.9167	1.58	612	205256±83324	0.93±0.48	0.58~1.74	1.05±0.36	0.67~1.58	1.02±0.20	0.83~1.25	0.218	0.540
212	2-Ethyl-furan	3208-16-0	3.8333	1.77	708	2060647±885528	1.20±0.34	0.85~1.68	1.08±0.55	0.65~2.01	0.72±0.16	0.47~0.87	0.000	0.821

213	2,4-Dimethylfuran	3710-43-8	4.0000	1.77	719	12732±7169	0.99±0.75	0.37~2.23	1.20±0.41	0.79~1.86	0.84±0.26	0.55~1.24	0.262	0.599
214	2-Propyl-furan	4229-91-8	5.0833	1.91	791	28424±11602	1.08±0.49	0.56~1.63	1.04±0.44	0.68~1.75	0.88±0.26	0.52~1.24	0.756	0.661
215	2-Ethyl-5-methyl-furan	1703-52-2	5.3333	1.94	806	51790±24592	1.31±0.47	0.86~1.89	1.07±0.39	0.79~1.75	0.61±0.15	0.49~0.83	0.000	1.015
216	2-n-Butyl furan	4466-24-4	7.0833	2.00	893	176795±59559	0.96±0.32	0.67~1.30	0.95±0.38	0.64~1.59	1.09±0.25	0.69~1.33	0.305	0.600
217	Pentyl-oxirane	5063-65-0	7.3333	2.08	906	6265±2617	1.12±0.21	0.83~1.41	0.73±0.11	0.62~0.87	1.17±0.35	0.74~1.69	0.004	1.118
218	2,7-Dimethyl-oxepine	1487-99-6	7.9167	2.19	935	49456±19130	0.98±0.36	0.66~1.39	1.09±0.38	0.85~1.72	0.93±0.22	0.74~1.25	0.462	0.745
219	2-Pentyl-furan	3777-69-3	9.0833	2.09	993	4331000±1184019	0.96±0.25	0.73~1.25	0.98±0.32	0.65~1.49	1.06±0.14	0.83~1.17	0.259	0.657
220	trans-2-(2-Pentenyl) furan	70424-14-5	9.3333	2.17	1006	224028±79939	0.99±0.32	0.67~1.35	1.03±0.36	0.71~1.59	0.99±0.22	0.74~1.23	0.828	0.649
221	Benzofuran	271-89-6	9.3333	2.66	1006	7047±6200	1.22±1.21	0.27~3.09	0.58±0.43	0.32~1.33	1.02±0.50	0.42~1.80	0.113	1.011
222	5-Ethyl-2-furaldehyde	23074-10-4	10.5000	2.82	1065	23408±12166	1.45±0.41	0.95~1.97	1.04±0.30	0.74~1.49	0.52±0.10	0.43~0.67	0.000	1.191
223	2-Hexyl-furan	3777-70-6	11.1667	2.08	1098	15780±7443	0.77±0.26	0.41~1.01	0.85±0.27	0.54~1.22	1.40±0.40	0.90~1.98	0.001	1.000
224	2,3-Dihydro-benzofuran	496-16-2	13.5000	2.93	1222	4551±2175	1.30±0.53	0.63~2.03	0.86±0.19	0.64~1.11	0.83±0.11	0.66~0.95	0.026	1.080
225	3-Phenyl-furan	13679-41-9	13.6667	2.89	1231	60308±21108	0.76±0.16	0.61~1.01	1.01±0.22	0.83~1.37	1.23±0.36	0.89~1.72	0.001	1.044
	**Nitrogen compounds**													
226	Pyrazine	290-37-9	4.2500	2.30	736	35029±21091	1.56±0.52	1.13~2.27	0.75±0.34	0.34~1.23	0.69±0.41	0.31~1.25	0.000	1.405
227	Pyridine	110-86-1	4.5000	2.26	753	93017±33251	1.29±0.13	1.12~1.49	1.02±0.26	0.77~1.40	0.69±0.14	0.52~0.89	0.000	1.103
228	1-Ethyl-1H-pyrrole	617-92-5	5.5833	2.27	819	40250±60946	1.92±2.48	0.44~6.28	0.75±0.53	0.31~1.62	0.33±0.20	0.14~0.65	0.000	0.850
229	Methyl-pyrazine	109-08-0	5.7500	2.48	827	45129±37623	1.81±0.98	0.96~3.47	0.62±0.19	0.36~0.81	0.57±0.18	0.41~0.88	0.000	1.448
230	2,5-Dimethyl-pyrazine	123-32-0	7.5000	2.51	915	28543±20865	1.69±0.92	0.80~3.23	0.52±0.16	0.37~0.78	0.79±0.10	0.63~0.90	0.000	1.614
231	2-Ethyl-6-methyl-pyrazine	13925-03-6	9.3333	2.53	1006	6381±6551	1.63±1.17	0.68~3.56	0.40±0.08	0.32~0.53	0.38±0.09	0.32~0.44	0.000	1.200
232	3-Ethyl-2,5-dimethyl-pyrazine	13360-65-1	10.9167	2.49	1085	5241±4531	1.49±0.90	0.64~2.86	0.38±0.08	0.26~0.48	nd	nd	0.000	1.244
233	2-Methyl-3-ethylmaleimide	20189-42-8	13.7500	3.03	1236	6232±3454	1.25±0.47	0.70~1.80	1.13±0.36	0.51~1.39	0.63±0.24	0.39~0.95	0.003	1.017
234	Indole	120-72-9	15.0000	3.54	1309	14021±9557	0.72±0.31	0.36~1.13	0.89±0.72	0.41~2.16	1.39±0.78	0.49~2.32	0.040	0.830
235	Caffeine	58-08-2	23.2500	4.45	1873	40319±16319	1.07±0.14	0.94~1.29	0.93±0.09	0.79~1.01	1.00±0.17	0.75~1.18	0.617	0.465
	**Sulfur compounds**													
236	Dimethyl sulfide	75-18-3	2.5000	1.49	526	4379232±1690184	1.11±0.30	0.84~1.57	1.14±0.25	0.82~1.49	0.76±0.24	0.50~1.01	0.008	0.772
	**Other compounds**													
237	Phenol	108-95-2	8.8333	2.65	981	13028±6127	1.23±0.38	0.85~1.66	0.77±0.20	0.61~1.11	1.00±0.44	0.46~1.56	0.018	1.212
238	Benzothiazole	95-16-9	13.8333	3.42	1241	4988±2852	0.92±0.20	0.68~1.12	1.37±0.77	0.61~2.46	0.63±0.23	0.46~1.03	0.002	1.460
	**Total**					91332369±11656416	1.06±0.12	0.91~1.19	0.98±0.08	0.90~1.07	0.95±0.06	0.90~1.05		

Note: [1] The data was shown as the mean value ± standard deviation; *: the compounds were identified by authentic standards; nd: not detected; BHYZ: Baihaoyinzhen; BMD: Baimudan; SM: Shoumei. [2] The ratio was calculated by the formula of A/A_{aver}, A represents the peak area of volatile compound in an individual sample, A_{aver} represents the average peak area of volatile compound in all samples. [3] The P-Value was calculated by Tukey s-b(K) test.

Table S3. The detailed performance of GC-O analysis of Baihaoyinzhen.

NO.	Compounds	Assessor 1		Assessor 2		Assessor 3		Assessor 4		Assessor 5	
		Aroma characteristics	AI[1]	Aroma characteristics	AI[1]	Aroma characteristics	AI[1]	Aroma characteristics	AI	Aroma characteristics	AI
1	Hexanal	Green, grassy	2	Cucumber-like, fresh	3	Green	1	Grassy	1	Grassy, green	3
2	Unknown 2	Pleasant	3			Floral	1	Floral, sweet	1	Coffee-like	2
3	Unknown 3	Milky, sweet	1	Vanilla-like	3			Popcorn-like	1	Slightly burnt	2
4	Unknown 4	Roasted, sweet	2					Burnt, sweet	3	Pollen-like	3
5	Unknown 5	Sweet, cream-like	3	Roasted sweet potato	3			Grain, glutinous rice	2	Roasted, lightly sauced	3
6	Unknown 6	Pesticide-like	1	Capsule-like	2	Capsule-like, powder-like	1				
7	Unknown 7	Pesticide-like	2	Metal	3			Sour-like	1	Sauced bean, salty	3
8	1-Octen-3-ol	Mushroom-like	2			Mushroom-like	2	Grassy	1	Green, rusty	2
9	Unknown 8	Tobacco-like, irritating	2	Metal, slightly floral	2	Rose-like	3	Wood, rusty	3	Rusty	3
10	Unknown 9	Stinky	2	Stinky	1			Stinky	3		
11	β-Cymene	Rice-like	1			Floral, fruity	1			Rice-like	2
12	Benzyl alcohol	Hay-like	2	Grassy, medicine-like	2	Fruity	1	Floral	2	Sweet, spicy	3
13	Benzeneacetaldehyde			Strong grassy aroma	3	Rose-like	3	Rose-like	3	Pollen-like	3
14	Unknown 10	Milky	2	Vanilla, coffee-like	3			Burnt, nutty	3	Caramel-like	2
15	tras-Linalool Oxide (furanoid)	Sweet	1	Floral, green	1					Coffee, caramel-like	3
16	3,5-Octadien-2-one isomer 2	Dried straw-like	2	Chinese medicine-like	3	Herbal medicine-like	2			Green, fresh	3
17	Linalool	Sweet, floral[2]	3	Sweet, honey	3	Floral	2	Grassy, fruity	3	Fresh, sweet[2]	3
18	Nonanal	Sweet, floral[2]	3	Strong sweet honey	3	Green, floral	3	Floral, Citrus, sweet	3	Fresh, sweet[2]	3
19	Phenylethyl Alcohol	Floral	2	Rose-like	3	Slightly floral	1	Rose-like	4	Floral, sweet	3
20	Unknown 12	Hay-like	1					Cucumber-like, green	2	Toothpaste-like, fresh	3
21	5-Ethyl-6-methyl-3-hepten-2-one	Stinky	2	Grassy, stinky	3	Stinky	2			Stinky	3
22	3-Nonen-1-ol, (E)-	Raw melon-like	2	Grassy aroma	4	Grassy, stinky	3	Cucumber, chocolate-like	3	Cucumber, coffee-like	3
23	2-Nonenal, (E)-			Stinky, ink-like	3	Plastic	2	Stinky, chocolate-like	4	Cucumber-like	3
24	1-Nonanol			Grassy	2			Cucumber-like, green	2	Cucumber-like, fresh	2
25	Naphthalene	Capsule-like	1	Fatty, bitter	2	Powder-like, non-pleasant	2			Irritating	3
26	Terpineol			Green, sweet	2	Green, floral	1			Green, fresh	3
27	Unknown 14			Licorice-like, metal	3	Powder-like	1	Non-pleasant	1		
28	cis-3-Hexenyl isovalerate	Star anise-like	1	Sweet, srong grassy aroma	3	Fresh, floral	2	Green, Cucumber-like	2	Complex odor, slightly	3
29	Geraniol	Slightly floral	1	Floral	3	Floral	2	Floral, rose-like	3	Slightly fresh	2
30	γ-Nonalactone	Slightly sweet	1	Cream-like, sweet	3	Sweet, floral	2	Cream-like, sweet	2	Sweet	2
31	Unknown 17					Irritating	1	Non-pleasant	2	Irritating	2
32	Unknown 18			Sweet, floral	2			Sweet, caramel-like	2	Honey	3
33	trans-β-Ionone			Sweet, floral	3	Cream-like, pleasant	2				
34	cis-Calamenene			Minty, toothpaste-like	2	Citrus pell-like	2	Grassy	1		

Note: [1] AI represents aroma intensity; [2] two aroma compounds were smelled continuously.

Table S4. The detailed performance of GC-O analysis of Baimudan.

NO.	Compounds	Assessor 1		Assessor 2		Assessor 3		Assessor 4		Assessor 5	
		Aroma characteristics	AI[1]	Aroma characteristics	AI[1]	Aroma characteristics	AI[1]	Aroma characteristics	AI[1]	Aroma characteristics	AI[1]
1	Hexanal			Grassy, sweet	3	Grassy	1	Grassy	3	Green	4
2	Unknown 2					Floral	2	Roasted	4	Coffee-like	3
3	Unknown 4	Sweet	2					Strong floral	4	Pollen-like	4
4	Unknown 5	Roasted, Sweet	1	Roasted sweet potato	3					Roasted bean-like	4
5	Unknown 7	Reagent or pesticide-like	1	Pesticide-like	2					Irritating	4
6	1-Octen-3-ol	Mushroom-like	1			Mushroom-like	3	Floral	3	Green, rusty	3
7	Unknown 8	Tobacco-like, irritating	2	Nail polish-like	3			Pesticide-like	4		
8	Benzyl alcohol			Chinese medicine-like	2	Rose-like	3	Green lemon-like	4	Pollen-like, fresh	3
9	Benzeneacetaldehyde	Green	2	Grassy, sweet	3	Rose-like	3	Green lemon-like	4	Pollen-like, sweet	4
10	2-Octenal, (E)-	Stinky	2	Stinky	2	Green	1			Sauce-like	3
11	Unknow 11			Non-pleasant, capsule-like	3	Non-pleasant	2	Non-pleasant	4		
12	tras-Linalool Oxide (furanoid)	Roasted, sweet	1			Floral	1	Floral	3	Roasted sweet potato	3
13	3,5-Octadien-2-one isomer 2	Hay-like	2			Pleasant, milky	4			Sweet, fresh	3
14	Linalool	Floral, fruity[2]	3	Green, sweet, honey[2]	4	Floral	2	Milky[2]	4	Floral, fruity[2]	4
15	Nonanal	Floral, fruity[2]	3	Green, sweet, honey[2]	4	Floral	3	Milky[2]	4	Floral, fruity[2]	4
16	Phenylethyl Alcohol	Floral	1	Floral	3			Floral, strong	3	Floral, sweet	3
17	5-Ethyl-6-methyl-3-hepten-2-one	Star anise-like	3	Grassy, stinky	3					Fresh, sweet	3
18	3-Nonen-1-ol, (E)-	Raw melon-like	2	Grassy, sweet	3	Grassy, stinky	3	Fruity, melon-like	4	Cucumber-like, fresh	4
19	2-Nonenal, (E)-	Cooked rice-like	1	Ink-like, fatty	3	Plastic	2			Cucumber-like, fresh	4
20	1-Nonanol					Fresh	1	Raw melon-like	2	Fresh	3
21	Unknown 13	Non-pleasant	1			Non-pleasant	3			Spicy	3
22	Naphthalene	Artemisia leaf-like	2	Chinese medicine-like	3	Powder-like, non-pleasant	2			Spicy	4
23	Terpineol			Leafy, sweet	3	Floral	2			Sweet	3
24	Unknown 14			Stinky, putridity-like	2	Non-pleasant	1	Sauce-like, salty	4	Sauced bean-like, salty	3
25	neral	Hay-like, pleasant	1			Floral, fruity	2			Floral	2
26	cis-3-Hexenyl isovalerate			Green, sweet, honey	3			Fresh, minty	4	Fresh, green	2
27	Geraniol	Floral	2	Fresh	3	Rose-like	3	Floral	3	Fresh, bean-like	2
28	Unknown 15	Floral	1			Floral, fruity	2			Floral, sweet	3
29	Unknown 16	Sweet, fruity, sour	1	Minty, citrus	1			Yogurt-like	4	Sweet, fruity, sour	2
30	γ-Nonalactone	Sweet, milky	2	Sweet, floral	1					Coconut-like	3
31	Unknown 17			Non-pleasant, plastic	2	Non-pleasant	1			Irritating	2
32	Unknown 18			Floral, rose-like	3			Caramel-like, sweet	4	Caramel-like	3
33	α-Ionone	Pleasant	1			Cream-like	1	Rose-like	3		
34	trans-β-Ionone			Sweet, floral, fruity	3	Cream-like, sweet	3				
35	cis-Calamenene	Toothpaste-like, minty	2	Minty	2	Fresh, floral	1	Fresh, minty	3	Fresh	3

Note: [1] AI represents aroma intensity; [2] two aroma compounds were smelled continuously.

Table S5. The detailed performance of GC-O analysis of Shoumei.

NO.	Compounds	Assessor 1		Assessor 2		Assessor 3		Assessor 4		Assessor 5	
		**Aroma characteristics**	**AI[1]**	**Aroma characteristics**	**AI[1]**	**Aroma characteristics**	**AI[1]**	**Aroma characteristics**	**AI[1]**	**Aroma characteristics**	**AI[1]**
1	Hexanal	Green	2	Grassy	3	Grassy	2	Grassy	3	Green, grassy	3
2	Unknown 1	Slightly grassy aroma	1			Fresh, green	2	Herbal medicine-like	4		
3	Unknown 3	Roasted, milky	2	Roasted	2			Roasted	2		
4	Uukonwn 4	Sweet	3					Floral	3	Pollen-like, sweet	3
5	Unknown 5	Roasted, sweet	3							sweet	3
6	Unknown 7	Pesticide-like	2	Grassy, Liquid	2	Stuffy, cooked	1			Sauce bean-like	2
7	1-Octen-3-ol	Mushroom-like	3			Mushroom-like	3	Green, fresh	3	Green, rusty	3
8	Unknown 8	Tobacco-like, irritating	3	Nail polish-like	3			Grassy or green	4	Rusty	3
9	Unknown 9	Stinky	2	Stinky	2			Stinky	4		
10	Benzyl alcohol			Chinese medicine-like	2	Rose-like	3			Green bean-like	2
11	Benzeneacetaldehyde	Grassy	1	Grassy	3	Rose-like	3	Green lemon-like	4	Pollen-like	3
12	2-Octenal, (E)-	Stinky	1	Stinky	3	Non-pleasant	1				
13	Unknown 10	Relatively pleasant	2					Roasted	2	Roasted	2
14	Unknown 11	Non-pleasant	2	Plastic, capsule-like	2	Capsule-like	2				
15	trans-Linalool oxide (furanoid)					Rice-like, milky	2	Fermented flour-like	3	Slightly burnt	3
16	3,5-Octadien-2-one isomer 2	Putrid grass-like	3	Raw peanut-like	3	Green, raw melon-like	3			Fresh, sweet	2
17	Linalool	Floral[2]	3	Rose-like, sweet[2]	4	Floral, fruity	3	Floral[2]	4	Milky	3
18	Nonanal	Floral[2]	3	Rose-like, sweet[2]	4			Floral[2]	4	Fresh, milky	3
19	Phenylethyl Alcohol	Floral, fruity	2	Floral	2	Floral	1			Specially floral	3
20	5-Ethyl-6-methyl-3E-hepten-2-one	Stinky	3	Grassy, stinky	2	Grassy, stinky	3			Fresh, stinky	3
21	3-Nonen-1-ol, (E)-	Raw melon-like, green	3	Green, floral	4	Grassy, stinky	4	melon-like	4	Cucumber-like, fresh	3
22	Naphthalene	Irritating	3	Ink-like, fatty	3	Powder-like, non-pleasant	3			Irritating	4
23	cis-3-Hexenyl butyrate	Cucumber-like	1			Cucumber-like	3			Fresh	3
24	Terpineol	Star anise-like	1			Fresh, floral	2			Fresh	3
25	cis-3-Hexenyl isovalerate	Star anise-like	2	Green, sweet	3	Fruity, floral	2	Floral, sweet	4	Fresh	2
26	Geraniol	Sweet, floral	3	Floral	3	Floral	3	Green lemon-like	4	Sweet	3
27	Unknown 18							Strongly sweet	4	Minty, strongly sweet	4
28	Unknown 19	Honey	1					Caramel-like	3	Sweet	2
29	α-Ionone	Soap-like	3			Milky, floral	2			Fresh, fruity, sweet	2
30	β-Ionone			Osmanthus flower-like	3	Cream-like, sweet	3				

Note: [1] AI represents aroma intensity; [2] two aroma compounds were smelled continuously.
